# Formation of Antiferromagnetic Spin Dimer Induced by Orientation Ordering of Water Molecule in Fluorinated Pincered Cu(II) Complexes

**DOI:** 10.1021/acs.inorgchem.6c01062

**Published:** 2026-09-11

**Authors:** Kazuhiro Nawa, Dongge Zheng, Katsuki Kinjo, Chun-Wai Tsang, Pin-Xiang Zeng, Gurumallappa Gurumallappa, Norman Lu, Taku J. Sato

**Affiliations:** † Institute of Multidisciplinary Research for Advanced Materials, 13101 Tohoku University, Sendai 980-8511, Japan; ‡ Institute of Organic and Polymeric Materials, 34877 National Taipei University of Technology, Taipei 10608, Taiwan (ROC); § Institute for Solid State Physics, University of Tokyo, Kashiwa 277-8581, Japan

## Abstract

Pincered transition metal complexes show rich crystal structures owing to intra- and intermolecular hydrogen bonding. Their structural diversity also motivates us to investigate their magnetic interactions mediated by hydrogen bonding. In this study, we report the structural and magnetic properties of copper mononuclear complexes 2FH-CuCl_2_·H_2_O and 3F-CuCl_2_·H_2_O, with a fluorinated pincered ligand. Both compounds are composed of a uniform chain of molecules coupled by O–H···Cl classical and improper hydrogen-bonding interactions. These compounds exhibit a structural transition induced by orientation ordering of water molecules, which drives bond alternation. The bond alternation also induces dimerization in magnetic interactions between nearest-neighbor Cu ions, evidenced by the magnetization following the isolated antiferromagnetic spin dimer model.

## Graphical abstract

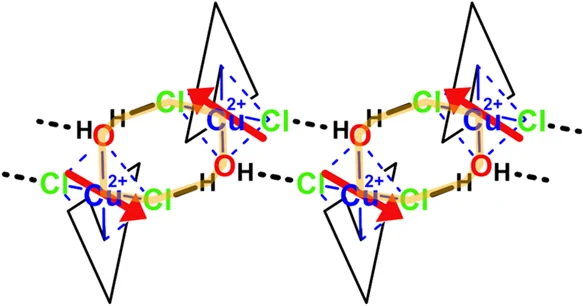

## Introduction

Molecular magnetic compounds are magnetic materials in which an individual molecule constitutes the fundamental magnetic unit. Their magnetic properties can be extremely tunable by chemical design, a level of control that is generally unattainable in conventional crystalline materials, owing to their large chemical diversity. In most molecular magnetic compounds, magnetic interactions are governed primarily by direct exchange between neighboring magnetic ions and superexchange mediated by ligand atoms. This makes molecular magnetic compounds an ideal experimental platform for systematically investigating the relationship between magnetic interactions and local structural parameters, such as bond lengths and bond angles. For example, comparative studies of Cu-based copper complexes have demonstrated that the magnetic interaction can change from ferromagnetic to antiferromagnetic as the metal–ligand–metal bond angle increases beyond approximately 95–98°, reflecting the competition and cancellation between direct exchange and superexchange contributions.^1,2^ This behavior is consistent with the Goodenough–Kanamori–Anderson rules,^3–6^ which predict ferromagnetic coupling for bond angles near 90° and antiferromagnetic coupling for angles approaching 180° between the same magnetic ions. Further studies based on ab initio calculations clarified the bond distance and bond angle dependence of magnetic interactions and clarified the presence of other factors decisive for magnetic interactions, such as the electronegativity of the coordinating atom in the terminal ligand.^7,8^ Understanding such magneto-structural correlations is essential not only for elucidating the magnetic properties of molecular magnets but also for providing fundamental insight into exchange mechanisms in crystalline magnetic materials.^9,10^

Beyond these conventional exchange pathways, there exist other classes of molecular magnetic compounds that do not involve simple metal–ligand–metal connections.^11^ One particularly intriguing example is molecular magnetic compounds coupled via hydrogen bonding. In most cases, a single magnetic coupling consists of a pair of hydrogen-bonding interactions, forming either a one-dimensional uniform chain or a molecular dimer.^12–25^ Remarkably, their magnetic interactions can exceed 100 K, despite the large Cu···Cu distances exceeding 5 Å.^22,23^ As a general trend, the stronger the hydrogen bonding, the smaller the separations between two coordination planes, leading to the enhancement of the overlap between the Cu d orbitals carrying an unpaired electron. Thus, the magnitude of the magnetic interaction increases with decreasing hydrogen-bond distances. Another example is π-π interactions between aromatic ligands.^26^ Although π–π interactions are a common source of magnetic interactions in organic or metal-organic radical-based compounds, they are much weaker and thus rarely found in molecular magnetic compounds with localized magnetic centers,^27–29^ ranging from sub-K to several K. Spin density on short contact atoms has been discussed as a major factor that contributes to the magnetic interactions.^27^

In this paper, we report the crystal structure and magnetic properties of fluorinated pincer-type Cu­(II) complexes 2FH-CuCl_2_·H_2_O and 3F-CuCl_2_·H_2_O, which are molecular magnetic compounds coupled by hydrogen-bonding interactions. The fluorinated pincer ligands consist of a central pyridine ring symmetrically substituted at the 2,6-positions with fluorinated alkyl groups, −CH_2_OR, R = CF_2_H (abbreviated as 2FH−), CF_3_ (3F−), and so on. High structural controllability of the molecular units can be achieved by varying the degree of fluorination of the alkyl groups, replacing halogen atoms within the alkyl moieties, and introducing halogen substitutions on the pyridine ring. Such modifications of the molecular units alter the intermolecular network and can switch the connectivity between two Cu ions responsible for spin-1/2 degrees of freedom. As discussed later, the fluorinated pincer-type Cu­(II) complexes exhibit both classical and nonclassical (improper) hydrogen-bonding-mediated connections of molecules. Their low-temperature crystal structures and magnetic properties reveal the formation of antiferromagnetic spin dimers induced by the orientation ordering of water molecules.

## Experiments

### General Procedures for Sample Synthesis

All chemicals, reagents, and solvents were commercially available and used as received without further purification. For example, 2,6-bis­(bromomethyl)­pyridine (CAS no.7703-74-4) and anhydrous copper­(II) chloride (CAS no. 7447-39-4). They were purchased from SynQuest (FL, USA) and Aldrich (Taipei, Taiwan), respectively. Gas chromatography–mass spectrometry (GC–MS) analyses were performed on an Agilent 6890 Series gas chromatograph equipped with a 5973-mass selective detector. Ligand syntheses were monitored using a Hewlett-Packard (HP) 6890 GC system fitted with a 30-m HP-1 capillary column (film thickness: 0.25-μm). The carrier gas flow rate was maintained at 1 mL/min with splitless injection. Electrospray ionization time-of-flight mass spectrometry (ESI–TOF–MS) data were collected using an Agilent 7890B GC system coupled with a 5977A mass selective detector. Infrared (IR) spectra were recorded on a PerkinElmer RX I FT–IR spectrophotometer. Phase purity was confirmed by powder X-ray diffraction experiments using a PANalytical Empyrean and a Rigaku SmartLab SE powder X-ray diffractometer with a Cu Kα radiation source. The collected powder X-ray diffraction patterns are shown in Figure S1.

### Synthesis of ((CF_2_HCH_2_OCH_2_)_2_py)­CuCl_2_·H_2_O [2FH-CuCl_2_·H_2_O] and ((CF_3_CH_2_OCH_2_)_2_py)­CuCl_2_·H_2_O [3F-CuCl_2_·H_2_O]

In a separate procedure, 2FH–Pin ligand (CF_2_HCH_2_OCH_2_)_2_py (0.37 mmol, 99 mg) and CuCl_2_ (0.37 mmol, 50 mg) were dissolved in 5 mL of anhydrous dichloromethane in a 50 mL round-bottom flask. The mixture was stirred at room temperature under a nitrogen atmosphere overnight. After completion, the solvent and volatiles were removed under reduced pressure. The crude product was washed with diethyl ether, affording the desired fluorinated ((CF_2_HCH_2_OCH_2_)_2_py)­CuCl_2._H_2_O as small blue crystalline materials. 3F-CuCl_2_.H_2_O was prepared in the same manner starting from the corresponding 3F–Pin ligand, (CF_3_CH_2_OCH_2_)_2_py, with the same molar amount.

Analytical data for 2FH-CuCl_2_·H_2_O: Yield: 80%. m.p.: >300 °C. FT-IR, ν (cm^–1^) = 3231, 3180 (water O–H stretching), 3069, 3023 (py-H stretching), 2958 (terminal CF_2_–H), 2924, 2877 (CH_2_ stretching), 1607 (py-C=C stretching), 1577 (py-C=N stretching), 1089, 1068 (CF_2_ stretching). HR-FAB (M–Cl-H_2_O, *m*/*z*): C_11_H_13_^35^Cl^63^CuF_4_NO_2_, calcd *m*/*z* 364.9867, found *m*/*z* 364.9867; C_11_H_13_^37^Cl^63^CuF_4_NO_2_, calcd *m*/*z* 366.9837, found *m*/*z* 366.9837; C_11_H_13_^35^Cl^65^CuF_4_NO_2_, calcd *m*/*z* 366.9849, found *m*/*z* 366.9849.

Analytical data for 3F-CuCl_2_.H_2_O: Yield: 82%; m.p.: 185–187 °C. FT-IR, υ (cm^–1^) = 3261, 3188 (OH stretching), 3074, 3041 (py-H stretching), 2952, 2924, 2855 (CH_2_ stretching), 1606 (py-C=C stretching), 1581 (py-C=N stretching) 1181, 1158, 1140, 1120, 1096 (CF_3_ stretching). HR-FAB, (M–Cl-H_2_O, *m*/*z*) *m*/*z* = C_11_H_11_^35^Cl^63^CuF_6_NO_2_: calcd *m*/*z* 400.9679, found *m*/*z* 400.9679; C_11_H_11_^37^Cl^63^CuF_6_NO_2_: calcd *m*/*z* 402.9649, found *m*/*z* 402.9649; C_11_H_11_^35^Cl^65^CuF_6_NO_2_: calcd *m*/*z*, found *m*/*z* 402.9660. Note: The HR-FAB is accurate to the right 4-places of decimal point, so its purity is considered as good.

### Single-Crystal X-ray Diffraction Studies of 2FH- and 3F-CuCl_2_·H_2_O

The single-crystal 2FH-CuCl_2_·H_2_O and 3F-CuCl_2_·H_2_O data were collected using a Rigaku XtaLAB Synergy R diffractometer equipped with a HyPix-Arc 150 detector and a rotating-anode X-ray source with Cu Kα radiation (λ = 1.54178 Å). Data were collected using ω scans and processed with CrysAlis PRO. A multiscan absorption correction was applied using CrysAlis PRO. The structure was solved using SHELXT and refined by full-matrix least-squares on *F* ^2^ using SHELXL.

### Single-Crystal Neutron Diffraction Studies of 3F-CuCl_2_·H_2_O

The temperature dependence of the nuclear reflections was investigated by using the neutron triple-axis spectrometer 4G-GPTAS in JRR-3.^30^ Neutron diffraction experiments were performed using neutrons with a wavelength of 2.358 Å and the collimations of 40′-80′-sample-80′-open. The higher-harmonic neutrons were removed by the pyrolytic graphite filter set before the monochromator. A single crystal of 3F-CuCl_2_·H_2_O with the size of 1 × 2 × 3 mm^3^ was used for the measurements. The crystal axes were confirmed by a 4-circle X-ray diffractometer (Huber) equipped with an in-house rotating-anode X-ray generator (Mo Kα radiation, Rigaku RA MultiMax9). Then, the sample was mounted on an aluminum plate using an aluminum foil so that the orthorhombic 0*KL* plane corresponds to the scattering plane of the spectrometer. The sample was sealed in an aluminum sample can with the He exchange gas. The can was attached to the cold head of the ^4^He closed-cycle refrigerator with the base temperature of 2.5 K.

### Magnetization Measurements of 2FH-CuCl_2_·H_2_O and 3F-CuCl_2_·H_2_O

The magnetization of 2FH-CuCl_2_·H_2_O and 3F-CuCl_2_·H_2_O was measured using Magnetic Property Measurement System (Quantum Design). For 2FH-CuCl_2_·H_2_O, an aggregate of small single-crystalline samples with 66.97 mg was used. The sample was set in a small capsule to perform magnetization measurements. For 3F-CuCl_2_·H_2_O, magnetization measurements were performed on a single-crystalline sample used for the neutron diffraction experiments. The sample weight was measured every time before the measurement since very small pieces of the sample can be cleaved from the largest piece. The weight was measured as 14.85, 14.98, and 14.89 mg for the measurements under *B*//*a*, *b*, and *c*, respectively, in the orthorhombic setting. The crystal axes of the sample were confirmed by the 4-circle X-ray diffractometer. Then, the sample was aligned on a plastic plate and inserted into a plastic straw. These samples were installed in the sample chamber of the MPMS at a stable temperature of 300 K and then cooled down to 10 K at a rate of 10 K/min before the measurement to suppress the cleavage of the sample due to a structural transition.

### DFT Calculations

To clarify the exchange pathways of the magnetic interactions, DFT calculations were performed using the ORCA software suite.^31–38^ Molecular orbitals of isolated 2FH-CuCl_2_·H_2_O and 3F-CuCl_2_·H_2_O molecules were calculated using the geometries determined at 100 K by single-crystal X-ray diffraction without optimizing the hydrogen positions. In addition, molecular orbitals, spin density distributions, and expected magnetic interactions were calculated for representative pairs of 2FH-CuCl_2_·H_2_O or 3F-CuCl_2_·H_2_O molecules. Single-point unrestricted Kohn–Sham DFT calculations were subsequently performed at the B3LYP-D3BJ/def2-TZVP level^35,36,39–42^ to describe the open-shell Cu^2+^ centers and the corresponding high-spin and broken-symmetry spin configurations. Total energies were obtained using VeryTightSCF convergence criteria and the DEFGRID3 numerical integration grid. The broken-symmetry spin states^43,44^ were generated to estimate the magnetic exchange interactions, and the isotropic exchange constants were evaluated using Yamaguchi’s approach.^45^

## Results

### Crystal Structure of Fluorinated Pincer Complex at Room Temperature

Single-crystal X-ray diffraction experiments revealed the formation of a six-coordinate Cu­(II) mononuclear complex. The crystallographic data and refinement parameters are listed in Table 1. 2FH-CuCl_2_·H_2_O crystallizes in the monoclinic space group of *C*2/*c* at 298 K, while it crystallizes in the monoclinic space group of *P*2_1_/*c* at 100 K. 3F-CuCl_2_·H_2_O crystallizes in the orthorhombic space group of *Pbcn* at 298 K, while it crystallizes in the monoclinic space group of *P*2_1_/*c* at 100 K. Since the molecular and crystalline structures of 2FH-CuCl_2_·H_2_O and 3F-CuCl_2_·H_2_O are similar, we focus on those of 3F-CuCl_2_·H_2_O in the main text and refer to those of 2FH-CuCl_2_·H_2_O in the Supporting Information.

**Table 1:** Crystallographic Data and Refinement Parameters for Fluorinated Pincer Cu Complexes

crystal data				
chemical formula	C_11_H_15_Cl_2_CuF_4_NO_3_ (2FH-CuCl_2_·H_2_O)		C_11_H_13_Cl_2_CuF_6_NO_3_ (3F-CuCl_2_·H_2_O)	
*M*_r_	419.68		455.66	
temperature (K)	100	298	100	298
crystal system	monoclinic	monoclinic	monoclinic	orthorhombic
space group	*P*2_1_/*c*	*C*2/*c*	*P*2_1_/*c*	*Pbcn*
*a*, *b*, *c* (Å), β (°)	7.3687(1), 11.0819(1), 18.7060(2), 95.5290(10)	19.5906(3), 11.1347(2), 7.5322(1), 107.2160(20)	7.9060(1), 19.1828(3), 10.7809(2), 91.9960(10)	19.2880(3), 10.8264(2), 8.1326(1), 90
*V* (Å^3^)	1520.41(4)	1569.42(5)	1634.03(4)	1698.25(5)
*Z*	4	4	4	4
μ (mm^–1^)	5.795	5.614	5.635	5.422
ρ (g/cm^3^)	1.833	1.776	1.852	1.782
crystal size (mm)	0.6 × 0.4 × 0.2		0.8 × 0.5 × 0.3	
Data Collection				
diffractometer	XtaLAB Synergy R, DW system, HyPix-Arc 150			
radiation type	Cu Kα (λ = 1.54178 Å)			
θ_max_	75.495	75.183	72.905	73.226
no. of measured, independent and observed reflections^a^	14265, 3067, 2971	7346, 1566, 1473	10959, 3168, 3014	11339, 1673, 1434
Refinement				
absorption corr.	multiscan (*CrysAlis PRO* 1.171.42.51a (Rigaku Oxford Diffraction, 2022))			
*hkl* range	–7 ≤ *h* ≤ 9	–24 ≤ *h* ≤ 24	–9 ≤ *h* ≤ 9	–23 ≤ *h* ≤ 22
	–13 ≤ *k* ≤ 13	–13 ≤ *k* ≤ 13	–23 ≤ *k* ≤ 23	–13 ≤ *k* ≤ 11
	–23 ≤ *l* ≤ 23	–9 ≤ *l* ≤ 7	–13 ≤ *l* ≤ 9	–9 ≤ *l* ≤ 9
*R*(*F* ^2^)^b^, *w*R** (*F* ^2^), *S*	0.0219, 0.0626, 1.069	0.0359, 0.0904, 1.065	0.0251, 0.0707, 1.078	0.0359, 0.0996, 1.100
no. of reflections	3067	1566	3168	1673
no. of parameters^c^	201	104	219	113
H atom treatment	H atom parameters constrained			
e max, min (e Å^–3^)	0.383, −0.402	0.516, −0.612	0.431, −0.353	0.367, −0.373

a Observed: *I* > 2σ­(*I*).

b *F* ^2^ > 2σ­(*F* ^2^).

c No restraints.

Figure 1 shows the structure of the single Cu­(II) mononuclear molecule. Each Cu­(II) ion forms a distorted octahedral coordination. A single Cu­(II) ion is coupled to one trifluorinated pincered [(CF_3_CH_2_OCH_2_)_2_-py] (3F-Pin) ligand, which consists of a central pyridine ring symmetrically substituted at 2,6-positions with fluorinated ether (−CH_2_OCH_2_CF_3_). At 298 K, two ethereal oxygen atoms are coordinated to the Cu­(II) ion via a Cu–O bond with a bond length of 2.468 Å, which is slightly larger than 2.427 Å in the 5F-Pin Cu­(II) complex.^46^ In addition, a pyridine nitrogen atom of a 3F-Pin ligand forms a Cu–N bond with a bond length of 1.990 Å, which is quite close to 2.009 Å of the 5F-Pin Cu­(II) complex.^46^ Two isolated chlorine atoms and one water molecule are also coupled to the Cu­(II) ion. The Cu–Cl and Cu–OH_2_ bond lengths are 2.284 and 1.956 Å, respectively. These are comparable with the Cu–Cl and Cu–OH_2_ bond lengths of 2.287 and 1.947 Å in CuCl_2_·2H_2_O, respectively.^47^ Among the six atoms coordinated to the Cu­(II) ion, the bond distance to the two ethereal oxygen atoms (2.468 Å) is much larger than those of the others. This is because the two ethereal oxygen atoms are coordinated to the Cu­(II) ion at the axial positions, while the pyridine nitrogen atom, one water molecule, and two chlorine atoms exist in the equatorial plane. The Jahn–Teller distortion stabilizes d^9^ electron configuration with one hole in the d*x*_2_-*y*_2_ orbital, which is extended within the equatorial plane. This orbital is responsible for intermolecular magnetic interactions, as we discuss later.

**Figure 1:**
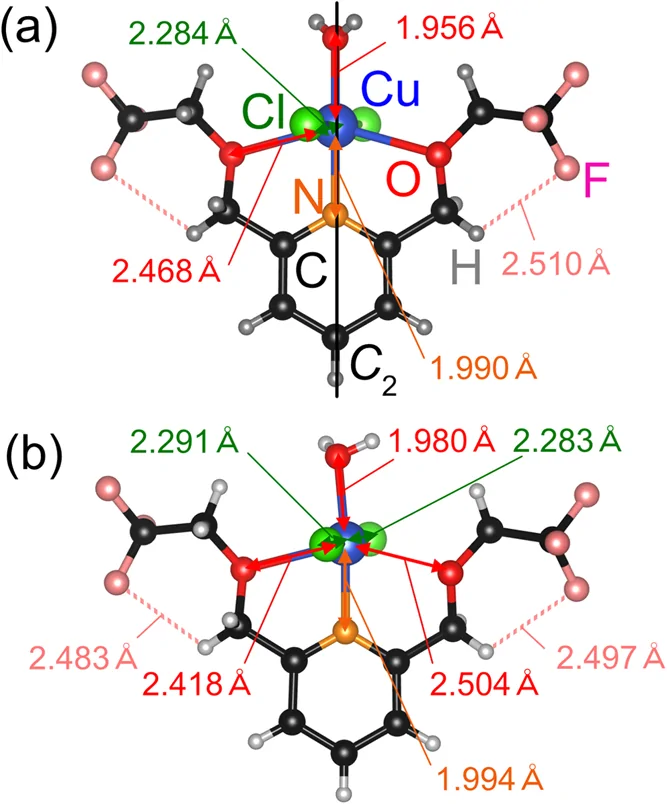
Single monomer molecule of 3F-CuCl_2_·H_2_O at (a) 298 K and (b) 100 K viewed perpendicular to the pyridine ring. The white, pink, black, orange, red, green, and blue circles represent H, F, C, N, O, Cl, and Cu atoms, respectively. The molecule is plotted using VESTA software suite.^72^

The molecular structure is also stabilized by bonding interactions between hydrogen and fluorine atoms, as indicated by the pink dashed lines in Figure 1. The bond length of 2.510 Å is slightly smaller than the sum of the van der Waals radii of hydrogen and fluorine atoms, 2.67 Å. The bonding promotes the formation of the H–C–O–C–C–F hexagonal ring and restricts rotation of a fluorinated ether group. All the bonds are related by a 2-fold rotational symmetry axis that passes through the pyridine nitrogen, copper, and water oxygen atoms, which is parallel to the *b*-axis. Along this axis, the Cu­(II) mononuclear molecules exhibit axial chirality.

The Cu (II) mononuclear molecules do not share any common atoms but are coupled by intermolecular hydrogen bonding interactions. Possible classical H···Cl hydrogen bonding between the nearest-neighbor Cu­(II) mononuclear molecules is shown as solid green lines in Figure 2a. The molecules are coupled via classical hydrogen bonding via a pair of O–H···Cl paths. The bond distances and angles are summarized in Table 2. The H···Cl and O–Cl distances are 2.206 and 3.054 Å, and the O–H···Cl bond forms a bond angle of 159.1°. These bond distances and angles are comparable with other copper complexes including the same type of classical hydrogen bonding, as listed in Table 2. The red-shifted O–H stretching bands observed in the IR spectra, appearing in the range of 3180–3216 cm^–1^, are consistent with hydrogen-bonding interactions. In addition, the O–Cl bond distance, slightly smaller than the sum of their van der Waals radii, suggests the formation of halogen bonding, as observed in other transition metal complexes.^48^

**Table 2:** Structural and Magnetic Properties of Copper Complexes Coupled by Hydrogen Bonding between Chlorine and Oxygen Ligands^a^

complex	2*J* (K)	Cu–Cu (Å)	possible exchange path	O–Cl (Å)	coordination plane distance	references
2FH-CuCl_2_·H_2_O (298 K)		5.332	O–H···Cl (×2)	3.010	2.356	this work
2FH-CuCl_2_·H_2_O (100 K)	–8.723	5.038	O–H···Cl (×2)	3.050	2.413	this work
		5.517		3.039	2.293	
3F-CuCl_2_·H_2_O (298 K)		5.434	O–H···Cl (×2)	3.054	2.393	this work
3F-CuCl_2_·H_2_O (100 K)	–11.34	5.099	O–H···Cl (×2)	3.069	2.589	this work
		5.584		3.070	2.244	
K(C_8_H_16_O_4_)_2_CuCl_3_·H_2_O	–29	6.000	O–H···Cl (×2)	3.136	0.790	^24^
H_2_L(CuCl_3_H_2_O)Cl (H_2_L = 1-(4′-pyridinium)pyridin-4-ol-ium)	–106	6.181	O–H···Cl (×2)	3.109	1.139	^25^

a The magnitude of the magnetic interactions estimated from the antiferromagnetic dimer model, the Cu···Cu distance, the possible exchange path, the O–Cl bond distance, and the distance between the coordination planes of the neighboring two copper monomers are listed.

**Figure 2:**
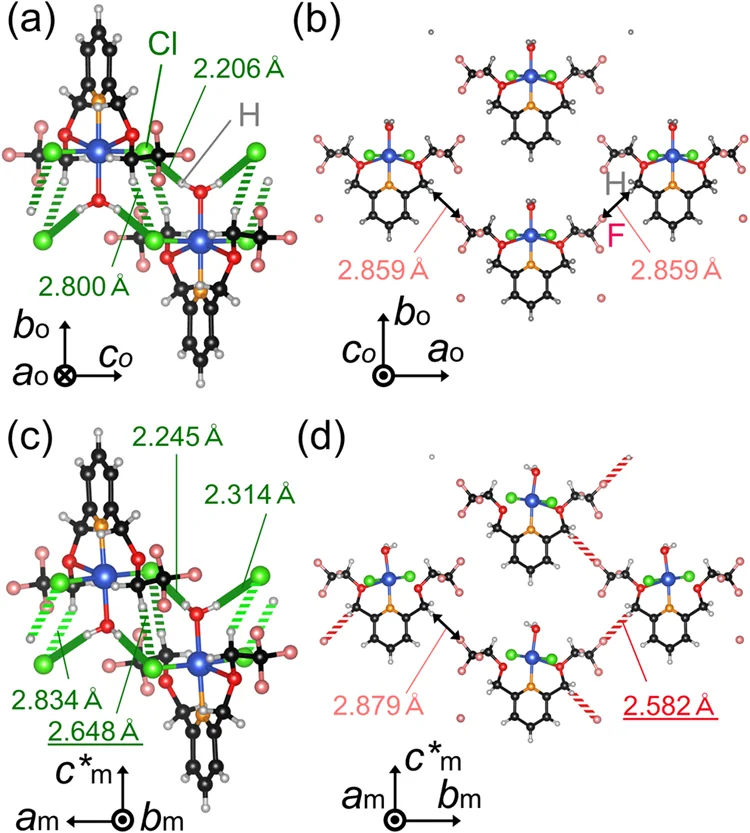
Intermolecular bonding in 3F-CuCl_2_·H_2_O at (a, b) 298 K and (c, d) 100 K. The structure is viewed along (a) the *a*-axis and (b) *c*-axis of the *Pbcn* structure and (c) *b*-axis and (d) *a*-axis of the *P*2_1_/*c* structure. The white, pink, black, orange, red, green, and blue circles represent H, F, C, N, O, Cl, and Cu atoms, respectively. Solid green lines represent H···Cl classical hydrogen bonding, while dashed green and red lines represent the H···Cl and H···F improper hydrogen bonding, respectively.

In addition, the strong electron-withdrawing character of a fluorinated ether group promotes improper (or nonclassical) hydrogen bonding. The improper hydrogen bonding is formed because of a delicate balance between hyperconjugative interactions and electrostatic interactions between inherent and induced electric dipoles.^49^ For instance, the intramolecular bonding between hydrogen and fluorine (dashed pink lines in Figure 1) is regarded as improper hydrogen bonding. The improper hydrogen bonding should also be decisive for the alternating stacking sequence of the molecules. The improper H···Cl hydrogen bonding is illustrated as dashed green lines in Figure 2a. A pair of improper hydrogen-bonding interactions are formed between nearest-neighbor molecules, with a bond distance of 2.800 Å. The distances are comparable with those of improper H···Cl hydrogen bonding observed in other binuclear transition metal complexes such as (5F-CuCl_2_)_2_ ^46^ and (2FH-ZnCl_2_)_2_.^50^

The symmetry relation between the molecules is shown in Figure 3a. The presence of the inversion symmetry between a pair of nearest-neighbor molecules leads to alternating sequences with opposite chirality along the *c*-axis. In addition, because of the 2-fold rotation symmetry, the H···Cl bonds connecting nearest-neighbor molecules become all symmetrically equivalent. As a result, a uniform chain of Cu­(II) ions with a bond distance of 5.434 Å is formed. The Cu···Cu distance is much larger than expected from commonly observed edge-shared geometry, such as 3.439 Å in (5F-CuCl_2_)_2_ ^46^ and 3.757 Å in CuCl_2_·2H_2_O.^47^ The local coordination of Cu atoms in 3F-CuCl_2_·H_2_O is almost the same as that in (5F-CuCl_2_)_2_, except for the presence of water molecules. The presence of water molecules significantly changes intermolecular interactions, leading to the formation of the zigzag Cu chain via hydrogen bonding, as illustrated in Figure 2a.

**Figure 3:**
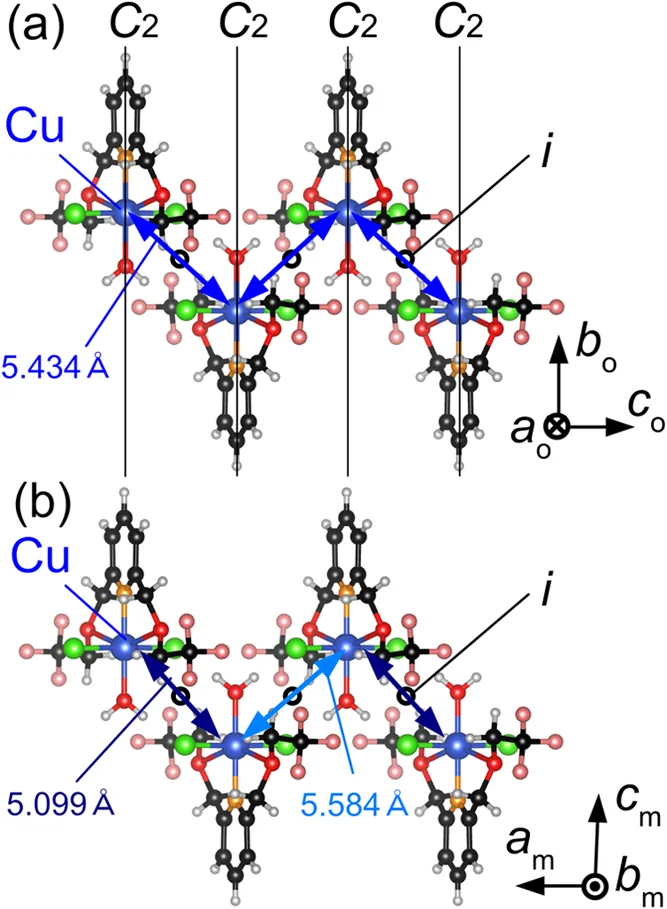
Symmetry operations in 3F-CuCl_2_·H_2_O for (a) the *Pbcn* structure at 298 K and (b) the *P*2_1_/*c* structure at 100 K. The structure is viewed along (a) the *a*-axis of the orthorhombic and (b) the *b*-axis of the monoclinic structures to show the coupling of Cu (II) ions. The white, pink, black, orange, red, green, and blue circles represent H, F, C, N, O, Cl, and Cu atoms, respectively.

### Crystal Structure of Fluorinated Pincer Complex at Low Temperatures

With decreasing temperature, the disappearance of the 2-fold rotation symmetry modifies the intramolecular and intermolecular structures. The six-coordinated Cu­(II) mononuclear molecule at 100 K is shown in Figure 1b. Most importantly, the orientation of the water molecules is changed from 298 K. The loss of the 2-fold rotation symmetry passing through the oxygen atom removes the restraint and allows the orientation ordering of the water molecule. In addition, due to the absence of the 2-fold rotation symmetry, the two Cu–O bonds coupled to the same Cu­(II) ion become inequivalent. The bond length of one bond decreases to 2.418 Å, while that of the other is 2.504 Å. The two Cu–Cl bonds coupled to the same Cu­(II) ion also have almost the same bond length, 2.283 and 2.291 Å. Since the change in the bond distances induced by the structural transition is small, the arrangement of the d*x*_2_-*y*_2_ orbital should not change so much with temperature.

The difference between 298 and 100 K is more prominent in the stacking sequence of molecules, as shown in Figure 2c,d. Because of the absence of 2-fold rotational symmetry, a single type of classical H···Cl hydrogen-bonding interaction splits into two inequivalent types, characterized by H···Cl bond lengths of 2.245 and 2.314 Å (Figure 2c). The symmetry lowering also makes a pair of improper H···Cl hydrogen-bonding interactions inequivalent; the H···Cl bond lengths become 2.648 and 2.834 Å for nearest-neighbor molecules (Figure 2c). In addition, the chains of molecules are further coupled by the H···F improper hydrogen bonding. At 298 K, the H···F bond, represented as dotted pink lines in Figure 2b shows a length of 2.859 Å. At 100 K, on the other hand, it becomes 2.582 and 2.879 Å (Figure 2d), and the former bond distance becomes smaller than the sum of the van der Waals radii of hydrogen and fluorine atoms. The H···Cl and H···F bond lengths of 2.648 and 2.582 Å, respectively (stressed by underline in Figure 2c,d), are much shorter than the 2.800 and 2.859 Å at 298 K. The large difference suggests that improper hydrogen bonding is also involved in the structural transition. As a result, dimerization of Cu pairs occurs in the uniform Cu chain with the Cu···Cu bond distances *d*_1_ = 5.099 Å and *d*_2_ = 5.584 Å at 100 K. The degree of the bond alternation is δ = 2­(*d*_2_ – *d*_1_)/(*d*_2_ + *d*_1_) = 0.09. This value is larger than that observed in other spin-1/2 one-dimensional antiferromagnets such as CuGeO_3_ (0.01),^51^ TiPO_4_ (0.03),^52^ and TiOCl (0.05).^53^ The large Cu···Cu distance may allow the high degree of bond alternation in 3F-CuCl_2_·H_2_O.

The main difference between the room- and low-temperature structure is the loss of the 2-fold rotation symmetry, due to the orientation ordering of the water molecule coupled to the Cu­(II) ions. While the water molecule is randomly oriented at 298 K, its orientation is ordered at 100 K to maximize the energy gain due to the classical and improper hydrogen bonding, leading to the dimerization of the Cu­(II) ions. The high transition temperature determined from the neutron diffraction experiments also supports that hydrogen-bonding interactions are involved in the structural transition. Figure 4 shows the omega scans of orthorhombic 002 or monoclinic 400 reflections at selected temperatures. A single peak splits into two peaks below *T*_s_ ∼ 260 K, indicating the formation of two monoclinic domains induced by the transition. The splitting angles gradually increase with decreasing temperature, suggesting the second-order nature of the transition. This is consistent with the space group of *P*2_1_/*c* at 100 K, which corresponds to the maximal subgroup of *Pbcn* induced by the loss of the 2-fold rotation symmetry. This structural transition is unique to mononuclear fluorinated pincered-type complexes, and has not been found in binuclear transition metal complexes, such as (5F-CuCl_2_)_2_ ^46^ and (2FH-ZnCl_2_)_2_,^50^ which do not contain water molecules.

**Figure 4:**
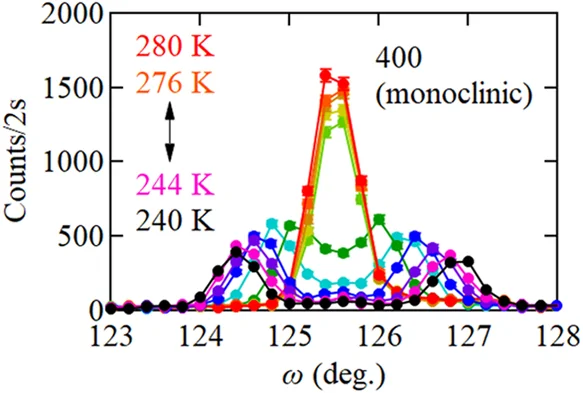
Omega scans of the monoclinic 400 reflection collected at different temperatures for a 3F-CuCl_2_·H_2_O.

### Magnetic Properties of Fluorinated Pincer Complexes

The dimerization of a pair of Cu atoms was also apparent in the magnetization measurement. Antiferromagnetic interactions between spin-1/2 carried by Cu­(II) were revealed from the temperature dependence of the magnetic susceptibility. Figure 5a shows the temperature dependence of the magnetic susceptibility of 3F-CuCl_2_·H_2_O under a magnetic field applied to the *a*_0_-, *b*_0_-, and *c*_0_-axes (the crystal axes for the room-temperature orthorhombic structure). At high temperatures, the magnetic susceptibility increases with a decreasing temperature, following the Curie–Weiss law. This can be verified by the linear temperature dependence of the inverse magnetic susceptibility, as shown in the inset of Figure 5a. The magnitude of the effective moment and the Weiss temperature were estimated from the Curie–Weiss fit between 30 and 250 K (below *T*_s_). The temperature-independent term χ_0_ was also included in the fitting function. The estimated parameters are listed in Table 3. The effective moment of 1.72–1.78 μ_B_ is comparable with or slightly larger than 1.73 μ_B_ expected for a spin-1/2 system, indicating the orbital contributions in the *g*-factor. The Weiss temperature of −3.7 to −3.3 K reveals the presence of weak magnetic interactions between Cu^2+^ ions. The intradimer interactions were not dependent on the magnetic field direction so much, suggesting the magnetic interactions are nearly isotropic. The fit yields the temperature-independent term χ_0_ of −1.4 to −0.5 × 10^–4^ cm^3^ mol^–1^. This value is consistent with the sum of the expected diamagnetic contributions estimated from Pascal constants^54–57^ (−2.1 × 10^–4^ cm^3^ mol^–1^) and the Van Vleck susceptibility of Cu^2+^ ions (typically +0.6 × 10^–4^ cm^3^ mol^–1^).

**Figure 5:**
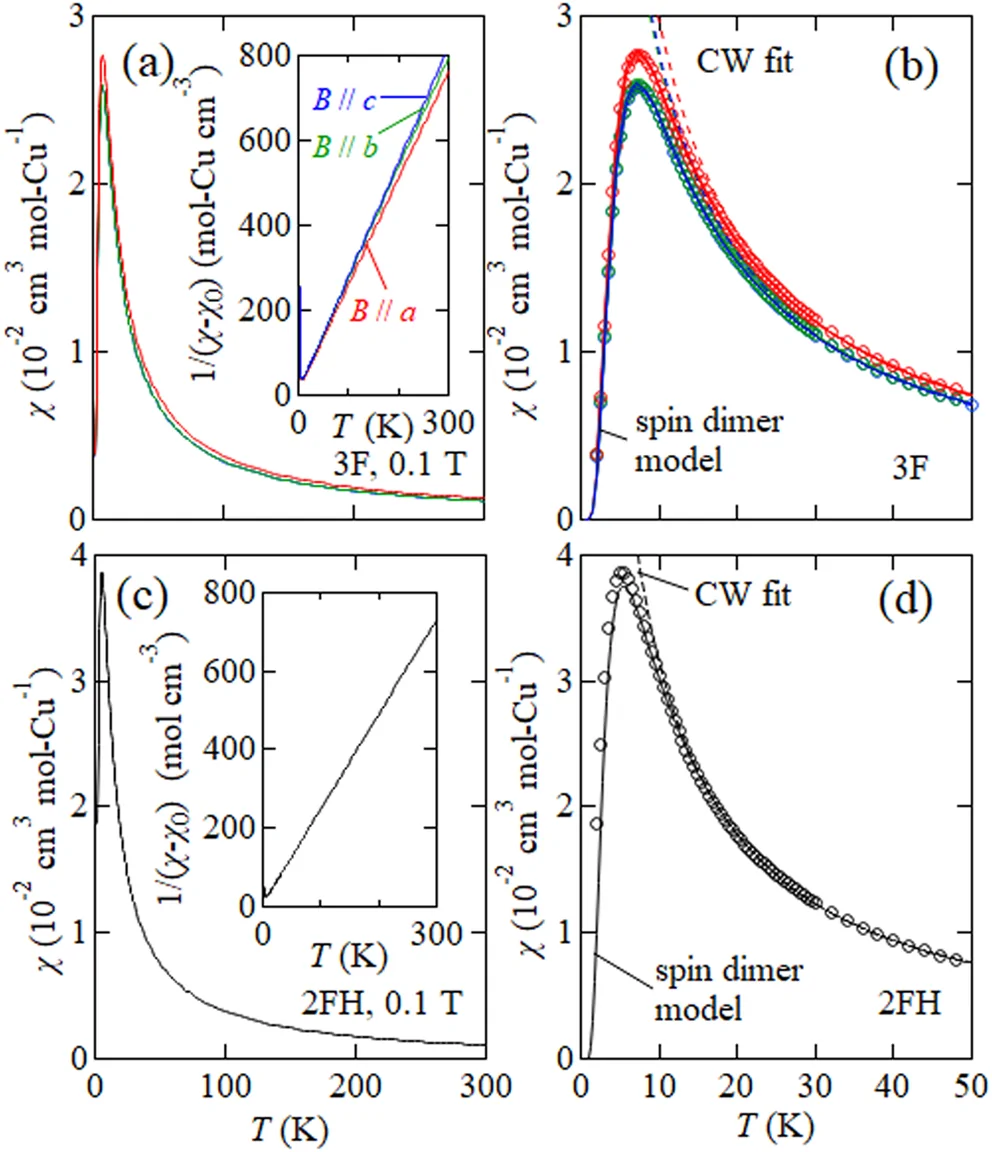
(a) Temperature dependence of the magnetic susceptibility of 3F-CuCl_2_·H_2_O for *B*//*a*_0_, *b*_0_, and *c*_0_. (inset) Temperature dependence of the inverse magnetic susceptibility. The contribution from the temperature-independent term χ_0_ estimated from the Curie–Weiss fit was removed. (b) Enlarged view of the magnetic susceptibility below 50 K. The dashed and solid curves represent the fit to the Curie–Weiss law and the antiferromagnetic spin dimer model, respectively. (c) Temperature dependence of the magnetic susceptibility and inverse magnetic susceptibility of polycrystalline 2FH-CuCl_2_·H_2_O and (d) the fit to the Curie–Weiss law and the antiferromagnetic spin dimer model.

**Table 3:** M and the Temperature-Independent Magnetic Susceptibility χ_0_ Estimated from the Curie-Weiss Fit between 30 and 250 K^a^

sample	2FH-CuCl_2_·H_2_O	3F-CuCl_2_·H_2_O		
field direction	polycrystalline	*B*//*a*_0_	*B*//*b*_0_	*B*//*c*_0_
μ_eff_	1.818	1.785	1.736	1.723
θ_W_ (K)	–2.589(2)	–3.405(5)	–3.705(3)	–3.331(2)
χ_0_ (10^–4^ cm^3^/mol)	–2.716(1)	–0.549(2)	–1.442(2)	–1.173(1)

a The error of the effective moment estimated from the fit is smaller than one-tenth of the last digit.

At low temperature, the magnetic susceptibility shows a broad peak at 7 K and decreases below 7 K irrespective of the magnetic field direction, as shown in Figure 5b. The temperature dependence indicates the occurrence of a singlet ground state, which is often observed in an antiferromagnetic spin dimer system. In an antiferromagnetic spin dimer, the magnetization is largely suppressed because of a spin singlet ground state, |*S*_tot_, *S*_tot,*z*_ > = |0, 0>. On the other hand, the magnetization increases with increasing temperature due to the contributions from thermally excited spin triplet states, |*S*_tot_, *S*_tot,*z*_ > = |1, 1>, |1, 0>, and |1, −1>. To confirm the presence of the antiferromagnetic spin dimer, the magnetic susceptibility was fit to an isolated antiferromagnetic spin dimer model.^58^ The fitting function is represented as

$$\chi =\frac{Ng^{2}\mu_{B}^{2}}{k_{B}T}\frac{e^{2J/k_{B}T}}{1+3e^{2J/k_{B}T}}+\chi_{0}$$

where *k*_B_ and μ_B_ represent the Boltzmann constant and the Bohr magneton, respectively. The *g*-factor *g* was treated as an adjustable parameter depending on the magnetic field direction, whereas the intradimer interactions 2*J* and the temperature-independent term χ_0_ were treated as adjustable parameters independent of the magnetic field direction. The fitting curves are shown as a solid curve in Figure 5b. The fit well reproduces the temperature dependence of the magnetic susceptibility for three different field directions. The parameters estimated from the fit are listed in Table 4. The intradimer interactions were estimated as 2*J* = −11.34 K. As discussed in the previous subsection, Cu­(II) ions form a chain with alternating bond distances along the monoclinic *a*-axis below 260 K. Thus, the isolated antiferromagnetic spin dimer model is too simplified to explain the whole model. However, the satisfactory fit indicates that antiferromagnetic interactions persist on one bond, forming an antiferromagnetic spin dimer, while antiferromagnetic interactions on the other bond become much weaker. Introduction of interdimer interactions slightly improves the fit at low temperatures (see Figure S5 for details). The result of the fit to an alternating chain model,^59^ where magnetic interactions of 2*J* and 2*J*′ are altered along the chain direction, is listed in Table 4. The fit yields a *J*′/*J* value of 0.233, supporting that interdimer antiferromagnetic interactions are present but weak.

**Table 4:** Magnitude of the *g*-Factor, Magnetic Interactions, and the Temperature-Independent Term Estimated from the Fit to the Isolated Antiferromagnetic Spin Dimer Model^57^ between 2 and 250 K^a^

sample	2FH-CuCl_2_·H_2_O		3F-CuCl_2_·H_2_O		
field direction	polycrystalline		*B*//*a*_0_	*B*//*b*_0_	*B*//*c*_0_
Isolated Antiferromagnetic Dimer Model					
*g*	2.091	2.048	1.977		1.974
2*J* (K)	–8.723(1)	–11.34(1)			
χ_0_ (10^–4^ cm^3^/mol)	–2.559(1)	0.11(16)			

Alternating Chain Model				
*g*	2.091	2.090	2.019	2.016
2*J* (K)	–7.067(1)	–10.49(2)		
*J*′/*J*	0.2996(2)	0.233(2)		
χ_0_ (10^–4^ cm^3^/mol)	–2.779(1)	–1.67(6)		

a The error of the g-factor estimated from the fit is smaller than one-tenth of the last digit.

The magnetization curve also supports the formation of the antiferromagnetic spin dimer. Since the excited state with |*S*_tot_, *S*_tot,*z*_ > = |1, 1> can be stabilized by the Zeeman energy under a magnetic field, the low-temperature magnetization curve should rapidly increase with an increasing magnetic field. The magnetization curve for *B*//*a* at 2, 20, and 60 K is shown in Figure 6a. At 2 K, the slope of the magnetization is suppressed below 2 T, while it largely increases above 4 T. Such a large increase was not observed at 20 and 60 K. The rapid increase of the low-temperature magnetization reflects that the excitation gap between the singlet ground state and the excited state with |*S*_tot_, *S*_tot,*z*_ > = |1, 1> gets smaller under a magnetic field. This expectation was confirmed by a fit to the isolated antiferromagnetic spin dimer model. The magnetization curve at three different temperatures and the three different magnetic field directions were fitted simultaneously by the following equation

$$M-M_{0}=\mathrm{Ng}\mu_{B}S\frac{2e^{2J/k_{B}T}\sinh\left(\frac{g\mu_{B}H}{k_{B}T}\right)}{1+e^{2J/k_{B}T}\left\{1+2\cosh\left(\frac{g\mu_{B}H}{k_{B}T}\right)\right\}}$$

using the *g*-factor of the three orthogonal directions and 2*J*. The estimated *g*-factor was 2.140 (*B*//*a*_0_), 1.988 (*B*//*b*_0_), and 1.967 (*B*//*c*_0_), which is consistent with the estimation from the magnetic susceptibility. The intradimer interactions 2*J* were estimated as −10.84(1) K, which is almost consistent with 2*J* = −11.34 K estimated from the temperature dependence of the magnetic susceptibility. The small deviation should be due to the presence of weak interdimer magnetic interactions, which are completely neglected in the model.

**Figure 6:**
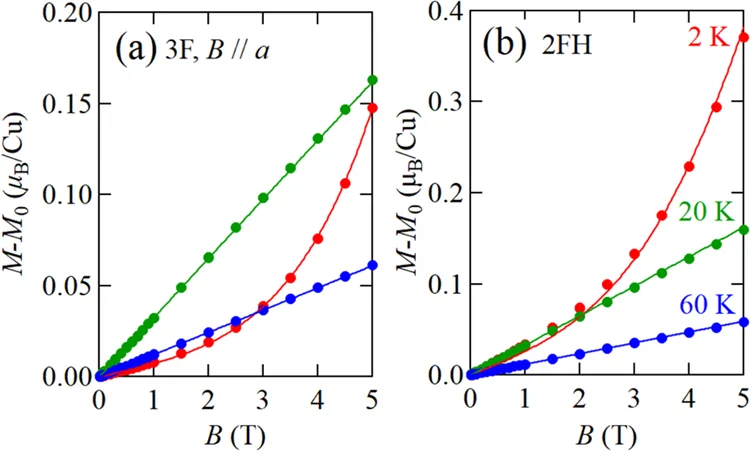
(a) Magnetization curve at 2, 20, and 60 K of single-crystalline 3F-CuCl_2_·H_2_O for *B*//*a*_0_. The contributions from the temperature-independent term χ_0_ estimated from the magnetic susceptibility were subtracted. The solid curves represent the fit to the isolated spin dimer model. (b) Magnetization curve of polycrystalline 2FH-CuCl_2_·H_2_O and the fit to the isolated spin dimer model.

Magnetic properties of 2FH-CuCl_2_·H_2_O are very similar to those of 3F-CuCl_2_·H_2_O, except for the small quantitative difference. The temperature dependence of the magnetic susceptibility (Figure 5c,d) shows a broad peak at 5 K, which is lower than the 7 K of 3F-CuCl_2_·H_2_O. The magnetization at 2 K, 5 T (Figure 6b) is 0.37 μ_B_, which is larger than 0.14 μ_B_ of 3F-CuCl_2_·H_2_O. These differences indicate weaker antiferromagnetic interactions than those of 3F-CuCl_2_·H_2_O. Indeed, the fit to the antiferromagnetic spin dimer model yields 2*J* of −8.723(1) K (from the magnetic susceptibility) and −8.09(1) K (from the magnetization curve), which is roughly 30% smaller than that of 3F-CuCl_2_·H_2_O.

### Molecular Orbital Responsible for Magnetic Interactions

DFT calculations provide microscopic support for a superexchange mechanism involving the Cu^2+^ d*x*_2_–*y*_2_-based orbitals. Figure 7a,b shows the isosurfaces of the occupied α-spin singly occupied molecular orbital (SOMO) and the corresponding unoccupied β-spin orbital for isolated 3F-CuCl_2_·H_2_O and 2FH-CuCl_2_·H_2_O molecules. These orbitals are predominantly derived from the Cu^2+^ d*x*_2_–*y*_2_ orbital and exhibit amplitudes on the pyridine N atom, the O atom of the coordinated water molecule, and the two Cl ligands. Small amplitudes are also observed on the pyridine ring, reflecting the contribution of the π-conjugated ligand framework.

**Figure 7:**
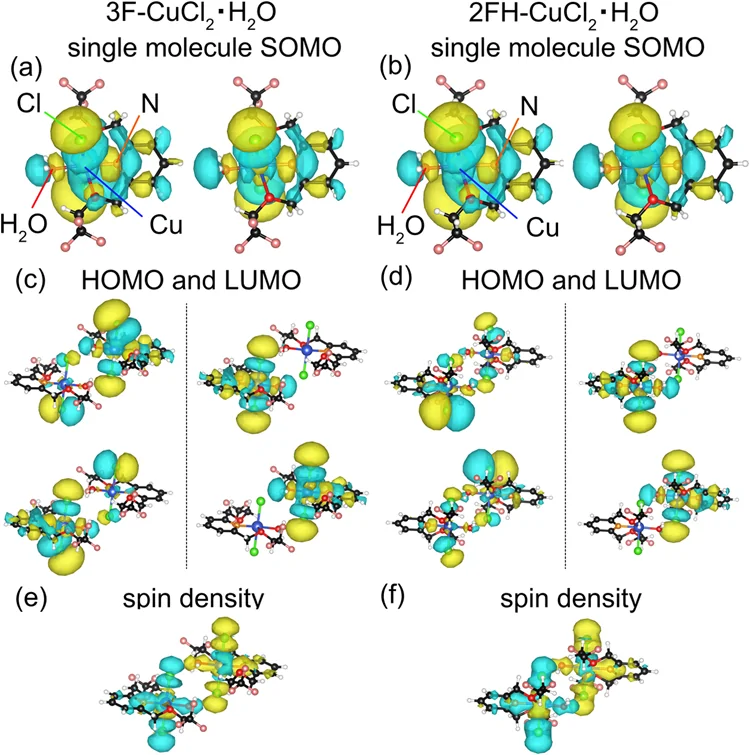
(a, b) Illustration of the occupied α-spin singly occupied molecular orbital (SOMO) and the corresponding unoccupied β-spin orbital for isolated (a) 3F-CuCl_2_·H_2_O and (b) 2FH-CuCl_2_·H_2_O molecules. (c, d) Illustration of two highest occupied molecular orbitals (HOMO) and two lowest unoccupied molecular orbitals (LUMO) of a pair of (c) 3F-CuCl_2_·H_2_O and (d) 2FH-CuCl_2_·H_2_O molecules that are coupled with antiferromagnetic interactions via a O–H···Cl path (“O–H···Cl” long in Table 5). (e, f) Illustration of spin density in the pair of (e) 3F-CuCl_2_·H_2_O and (f) 2FH-CuCl_2_·H_2_O molecules, which is the same as those in (c, d). An isosurface value of 0.01 au (a–d) and 0.0005 au (e, f) is used in the figure.

In addition, to investigate possible exchange paths, magnetic interactions and the relevant frontier molecular orbitals were estimated by DFT calculations. Table 5 summarizes the calculated exchange interactions for representative dimers where they are connected either through an O–H···Cl path or pyridine rings. For both 3F-CuCl_2_·H_2_O and 2FH-CuCl_2_·H_2_O, the largest antiferromagnetic interactions are found for the pair connected through the O–H···Cl bond with the larger distance. The frontier molecular orbitals of the antiferromagnetic dimer with the largest exchange interactions are shown in Figure 7c,d. This orbital has a finite contribution along the O–H···Cl path, suggesting the O–H···Cl hydrogen-bonding-assisted superexchange as a plausible origin of the sizable antiferromagnetic interaction between Cu^2+^ magnetic moments. Figure 7e,f illustrates the distribution of spin density on the pair of molecules. Large spin density is left on the O atom of the water molecule and the two Cl ligands, supporting the O–H···Cl bond as the superexchange path connecting the d*x*_2_–*y*_2_ orbital on the Cu atom.

**Table 5:** Magnetic Interactions Estimated from DFT Calculations Based on a Pair of 2FH-CuCl_2_·H_2_O or 3F-CuCl_2_·H_2_O Molecules^a^ ^,^ ^b^

bonds	O–H···Cl short	O–H···Cl long	stacked pyridine short	stacked pyridine long
2FH-CuCl_2_·H_2_O				
Cu–Cu distance (Å)	5.038	**5.517**	8.056	8.364
2*J* (K)	3.68	–**6.40**	0.89	–5.21
3F-CuCl_2_·H_2_O				
Cu–Cu distance (Å)	5.099	**5.584**	8.192	8.238
2*J* (K)	6.79	–**8.91**	0.92	–7.45

a Magnetic interactions are estimated for four representative bonds named “O–H···Cl short”, “O–H···Cl long”, “stacked pyridine short”, and “stacked pyridine long”. The corresponding bonds are illustrated in Figure 8.

b The bold values indicate the largest magnitude of 2J.

Slightly weaker antiferromagnetic interactions were also found between a pair of molecules with stacked pyridine rings. A finite contribution of the frontier molecular orbital suggests the possibility of weak magnetic interactions along the C–H···Cl path, despite the relatively large bond distance above 2.975 Å (see Figure S6). The above two antiferromagnetic interactions might effectively form the alternating chain, which is illustrated as the chain consisting of red solid and orange dashed lines along the diagonal direction in Figure 8.

**Figure 8:**
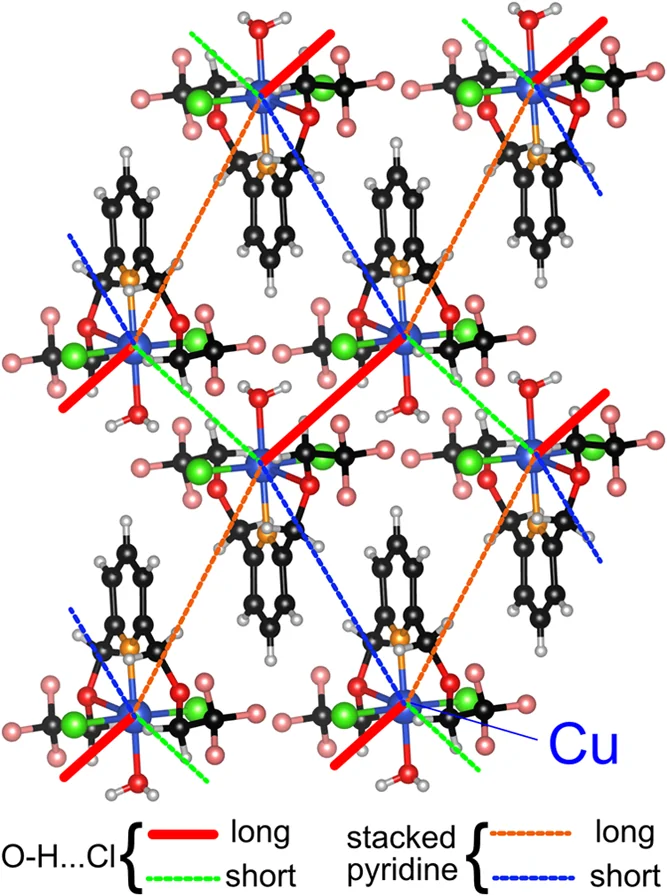
Possible exchange paths in 3F-CuCl_2_·H_2_O. The exchange paths via O–H···Cl bond are illustrated as red solid and green dashed curves (corresponding to “O–H···Cl long” and “O–H···Cl short” in Table 5, respectively). The exchange paths between molecules with stacked pyridine rings are illustrated as orange dashed and blue dashed curves (corresponding to “stacked pyridine long” and “stacked pyridine short” in Table 5, respectively).

## Discussion

The crystal structures and magnetic properties of 2FH-CuCl_2_·H_2_O and 3F-CuCl_2_·H_2_O reveal the presence of a structural transition associated with bond alternation, resulting in the formation of antiferromagnetically coupled spin dimers. The neighboring molecules do not share common atoms, and the Cu–Cu distances between adjacent molecules are sufficiently large that a few atoms must be involved in the magnetic exchange. These structural and magnetic properties are in contrast with those of (5F-CuCl_2_)_2_, which contains a well-defined structural dimer but does not exhibit clear evidence of antiferromagnetic spin dimer formation.^46^ In 2FH-CuCl_2_·H_2_O and 3F-CuCl_2_·H_2_O, the neighboring molecules are coupled by hydrogen bonding, which can mediate magnetic interactions. In addition, π-π interactions between pyridine rings of neighboring molecules could be possible. Usually, the former magnetic interactions are larger than those of the latter. Thus, it is likely that an antiferromagnetic dimer is formed via hydrogen-bonding-mediated paths, as has been reported for a number of Cu­(II) complexes.

Crystal structure and magnetic properties of copper complexes where magnetic interactions are mediated by the possible hydrogen bonding between chlorine and oxygen ligands are summarized in Table 2. Generally, the magnitude of the magnetic interactions mediated by hydrogen bonding ranges from a sub-K to a hundred K.^12–25^ Magnetic interactions become stronger with a decreasing hydrogen-bond distance and a decreasing interval between the coordination planes of the neighboring two copper monomers.^60^ The O­(−H)···Cl bond distance of 3.050, 3.039 Å in 2FH-CuCl_2_·H_2_O and 3.069, 3.070 Å in 3F-CuCl_2_·H_2_O (100 K) is comparable with other copper complexes that have the same type of the O­(−H)···Cl bond.^24,25^ On the other hand, the distance between the coordination planes of the neighboring two copper monomers, 2.413, 2.293 Å in 2FH-CuCl_2_·H_2_O and 2.589, 2.244 Å in 3F-CuCl_2_·H_2_O (100 K), is much larger than those of the other two compounds as listed in Table 2. The larger interplane distance may result in magnetic interactions of −9.4 K (2FH-CuCl_2_·H_2_O) and −11.8 K (3F-CuCl_2_·H_2_O) being much weaker than −29 and −106 K of the other two compounds. This discussion also suggests that Cu–Cu bonds with smaller interplane distance should be responsible for the formation of the antiferromagnetic dimer, though they have a larger bond distance of 5.517 Å (2FH-CuCl_2_·H_2_O) and 5.584 Å (3F-CuCl_2_·H_2_O). Overlap of molecular orbitals should become larger with decreasing interplane distance. Magnetic interactions estimated from the DFT calculations support the formation of an antiferromagnetic dimer in the same pair of Cu atoms. The pair forming an antiferromagnetic dimer could be further verified by inelastic neutron scattering experiments by observing intensity oscillations induced by the interference of the neutrons scattered by two Cu atoms forming antiferromagnetic dimers.^61,62^

On the other hand, a large bond distance between the short contact atoms on the pyridine rings suggests that π-π interactions are negligible in these compounds. For instance, in 3F-CuCl_2_·H_2_O, the short contact atoms form a bond distance of 3.563 or 3.850 Å, which is larger than the threshold value of ∼3.4 Å that can allow magnetic interactions via π-π interactions.^27–29^ The large bond distance indicates that π-π interactions between pyridine rings should be negligible. Instead of π-π interactions, the DFT calculations suggest the presence of magnetic interactions along the C–H···Cl path. Determining accurate positions of hydrogen atoms by neutron diffraction experiments is necessary to distinguish the exchange path more accurately by DFT calculations (see the Supporting Information for details).

The unique characteristics of the mononuclear fluorinated pincered-type complexes, 3F-CuCl_2_·H_2_O and 2FH-CuCl_2_·H_2_O, are evident from the different structural and magnetic properties compared with those of the binuclear transition metal complexes, such as (5F-CuCl_2_)_2_.^46^ The former compound exhibits antiferromagnetic dimer formation despite the nearly chain-like Cu arrangements. On the other hand, the latter compound does not show a clear signature of the antiferromagnetic dimer, though a single molecule consists of a pair of Cu atoms. This comparison highlights the distinctive nature of the mononuclear fluorinated pincer-type complexes, whose magnetic behavior is governed by sizable exchange interactions over long intermolecular distances.

Compared with other compounds that include hydrogen-bonding-mediated magnetic interactions, 2FH-CuCl_2_·H_2_O and 3F-CuCl_2_·H_2_O are unique in terms of the existence of the structural transition induced by the orientation ordering of water molecules. The structural transition triggers bond alternation, resulting in a Cu–Cu bond with moderate magnetic interactions and another Cu···Cu bond with negligible magnetic interactions in the same chain. Both Cu···Cu bonds are coupled by hydrogen bonding with similar bond distances and angles. The high structural controllability of fluorinated pincer-type Cu­(II) complexes would make it possible to investigate magneto-structural correlations in hydrogen-bonding mediated interactions: adjusting the intermolecular packing would be possible by increasing the degree of fluorination of the alkyl groups or introducing halogen substitutions on the pyridine ring. In addition, since these complexes contain rich noncovalent bonding between molecules as observed in (5F-CuCl_2_)_2_ ^46^ and (2FH-ZnCl_2_)_2_,^50^ it would be interesting to investigate if the noncovalent bonding can induce magnetic interactions between molecules.

## Conclusion

The formation of an antiferromagnetic dimer was found in the copper complex with a fluorinated pincered ligand 2FH-CuCl_2_·H_2_O and 3F-CuCl_2_·H_2_O. Single-crystal X-ray and neutron diffraction experiments revealed the presence of a uniform chain of copper atoms, coupled by a pair of O–H···Cl bonds. At low temperatures, the orientation ordering of water molecules triggers a structural transition accompanied by alternation in Cu–Cu bonds with bond lengths of 5.0–5.1 and 5.5–5.6 Å. The strong dimerization in a magnetic coupling is evidenced by the magnetization following the isolated antiferromagnetic spin dimer model. The small magnitude of the exchange parameters was understood in terms of the large distances between the coordination planes of the neighboring two copper ions. A high degree of structural controllability, together with the presence of a unique structural transition, would enable a systematic investigation of hydrogen-bonding-mediated magnetic interactions as a function of local structural parameters, and provide an excellent platform for realizing and testing a well-defined spin Hamiltonian.

## Supplementary Material



## Notes

Powder diffraction patterns of 2FH-CuCl_2_·H_2_O and 3F-CuCl_2_·H_2_O, molecular and crystal structure of 2FH-CuCl_2_·H_2_O, detailed estimation of magnetic interactions, and possible magnetic exchange path through pyridine rings (PDF)

## ∥

Department of Physics, The Chinese University of Hong Kong, Hong Kong, China

## References

[1] Hay P. J., Thibeault J. C., Hoffmann R. (1975). Orbital interactions in metal dimer
complexes. J. Am. Chem. Soc..

[2] Crawford V. H., Richardson H. W., Wasson J. R., Hodgson D. J., Hatfield W. E. (1976). Relation
between the singlet-triplet splitting and the copper-oxygen-copper
bridge angle in hydroxo-bridged copper dimers. Inorg. Chem..

[3] Anderson P. W. (1950). Antiferromagnetism.
Theory of Superexchange Interaction. Phys. Rev..

[4] Goodenough J. B. (1955). Theory
of the Role of Covalence in the Perovskite-Type Manganites [La, *M*(II)]­MnO3. Phys. Rev..

[5] Goodenough J. B. (1958). An interpretation
of the magnetic properties of the perovskite-type mixed crystals La1–xSrxCoO3−λ. J. Phys. Chem. Solids.

[6] Kanamori J. (1959). Superexchange
interaction and symmetry properties of electron orbitals. J. Phys. Chem. Solids.

[7] Ruiz E., Alemany P., Alvarez S., Cano J. (1997). Toward the Prediction
of Magnetic Coupling in Molecular Systems: Hydroxo- and Alkoxo-Bridged
Cu­(II) Binuclear Complexes. J. Am. Chem. Soc..

[8] Rodríguez-Fortea A., Alemany P., Alvarez S. (2002). Exchange Coupling in
Halo-Bridged Dinuclear Cu­(II) Compounds: A Density Functional Study. Inorg. Chem..

[9] Nawa K., Yajima T., Okamoto Y., Hiroi Z. (2015). Orbital Arrangements
and Magnetic Interactions in the Quasi-One-Dimensional Cuprates ACuMoO_4_(OH). Inorg. Chem..

[10] Mizuno Y., Tohyama T., Maekawa S., Osafune T., Motoyama N., Eisaki H., Uchida S. (1998). Electronic states and magnetic properties
of edge-sharing Cu-O chains. Phys. Rev. B.

[11] Atzori M., Serpe A. D., Deplano P., Schlueter J. A., Schlueter J. A., Mercuri M. L. (2015). Tailoring magnetic properties of
molecular materials through non-covalent interactions. Inorg. Chem. Front..

[12] Salunke-Gawali S., Rane S. Y., Puranik V. G., Guyard-Duhayon C., Varret F. (2004). Three dimensional hydrogen-bonding network in a copper
complex of 2-hydroxy-1,4-naphthoquinone: structural, spectroscopic
and magnetic properties. Polyhedron.

[13] Estes W. E., Hatfield W. E. (1978). Unusual Magnetic Properties in Two Copper­(II) Chelates
of Schiff Bases Derived from. alpha.-amino acids: a dimeric interaction
in a structural linear chain. Inorg. Chem..

[14] Plass W., Pohlmann A., Rautengarten J. (2001). Magnetic Interactions as Supramolecular
Function: Structure and Magnetic Properties of Hydrogen-Bridged Binuclear
Copper­(ii) Complexes. Angew. Chem., Int. Ed..

[15] Talukder P., Sen S., Mitra S., Dahlenberg L., Desplanches C., Sutter J.-P. (2006). Evidence for Hydrogen-Bond-Mediated Exchange Coupling
in an Aqua-Bridged CuII Dimer: Synthesis, Magnetic Study and Correlation
with Density Functional Calculations. Eur. J.
Inorg. Chem..

[16] Klein C. L., Majeste R. J., Trefonas L. M., Oconnor C. J. (1982). Magnetic properties
and molecular structure of copper­(II) complexes of pyrazinecarboxylic
acid. Inorg. Chem..

[17] Tang J., Costa J. S., Golobič A., Kozlevčar B., Robertazzi A., Vargiu A. V., Gamez P., Reedijk J. (2009). Magnetic Coupling
between Copper­(II) Ions Mediated by Hydrogen-Bonded (Neutral) Water
Molecules. Inorg. Chem..

[18] Hamaguchi T., Nagata T., Hayami S., Kawata S., Ando I. (2017). pH-Dependent
structural diversity of a 2-pyridinemethanol Cu complex and its relatively
strong magnetic exchange coupling via hydrogen bonding. Dalton Trans..

[19] Baran P., Boča R., Breza M., Elias H., Fuess H., Jorík V., Klement R., Svoboda I. (2002). The spectroscopic and
structural properties of copper­(II) complexes of the novel tridentate
(ONO) pyridine N-oxide ligand Hpoxap. Polyhedron.

[20] Okazawa A., Ishida T. (2009). Super–superexchange coupling through a hydrogen
bond in a linear copper­(II) complex, [Cu­(LH)­(L)]·BF_4_·2H_2_O (LH = N-tert-butyl-N-2-pyridylhydroxylamine). Chem. Phys. Lett..

[21] Muhonen H. (1986). Exchange Interaction
through Hydrogen-Bond Bridges and the Effect of a Single-Oxygen Bridge.
Crystal Structures and Magnetic Susceptibilities of Two Binuclear
Copper­(II) Complexes of 2-Amino-2-methyl-l-propanol. Inorg. Chem..

[22] Bertrand J. A., Black T. D., Eller P. G., Helm F. T., Mahmood R. (1976). Polynuclear
complexes with hydrogen-bonded bridges. Binuclear complex of N,N′-bis­(2-hydroxyethyl)-2,4-pentanediimine
with copper­(II). Inorg. Chem..

[23] Bertrand J. A., Fujita E., VanDerveer D. G. (1980). Polynuclear complexes with hydrogen-bonded
bridges. 4. Structure and magnetic properties of binuclear copper­(II)
complexes of amino alcohols. Inorg. Chem..

[24] van
Well N., Bolte M., Delley B., Wolf B., Lang M., Schefer J., Rüegg C., Assmus W., Krellner C. (2017). Interplay
between structure and magnetism in the low-dimensional spin system:
K­(C_8_H_16_O_4_)_2_CuCl_3_·H_2_O. CrystEngComm.

[25] Monroe J. C., Carvajal M. A., Deumal M., Landee C. P., Rademeyer M., Turnbull M. M. (2020). Revisiting the Role of Hydrogen Bonding in the Strong
Dimer Superexchange of a 2D Copper­(II) Halide Honeycomb-Like Lattice:
Structural and Magnetic Study. Inorg. Chem..

[26] Hunter C. A., Sanders J. K. M. (1990). The nature of π-π interactions. J. Am. Chem. Soc..

[27] Chi Y.-H., Shi J.-M., Li H.-N., Wei W., Cottrill E., Pan N., Chen H., Liang Y., Yu L., Zhang Y.-Q., Hou C. (2013). π–π Stacking, spin density and magnetic coupling. Dalton Trans..

[28] Venegas-Yazigi D., Brown K. A., Vega A., Calvo R., Aliaga C., Santana R. C., Cardoso-Gil R., Kniep R., Schnelle W., Spodine E. (2011). Exchange Interactions Through π–π
Stacking in the Lamellar Compound [{Cu­(bipy)­(en)}­{Cu­(bipy)­(H2O)}­{VO3}­4]­n. Inorg. Chem..

[29] Ramirez
de Arellano C., Escriva E., Gomez-Garcia C. J., Minguez Espallargas G., Ballesteros R., Abarca B. (2013). CrystEngComm.

[30] Nawa K., Okuyama D., Wu H., Murasaki R., Matsuzaka S., Kinjo K., Sato T. J. (2024). Present Status and Future Prospects
of the Thermal-neutron Triple-axis Spectrometer 4G-GPTAS. J. Phys. Soc. Jpn..

[31] Neese F. (2025). Software update:
the ORCA program system, version 6.0. WIRES
Comput. Mol. Sci..

[32] Neese F. (2003). An improvement
of the resolution of the identity approximation for the formation
of the Coulomb matrix. J. Comput. Chem..

[33] Neese F. (2004). Definition
of corresponding orbitals and the diradical character in broken symmetry
DFT calculations on spin coupled systems. J.
Phys. Chem. Solids.

[34] Neese F., Wennmohs F., Hansen A., Becker U. (2009). Efficient, approximate
and parallel Hartree-Fock and hybrid DFT calculations. A ‘chain-of-spheres’
algorithm for the Hartree-Fock exchange. Chem.
Phys..

[35] Grimme S., Antony J., Ehrlich S., Krieg H. (2010). A consistent and accurate
ab initio parametrization of density functional dispersion correction
(DFT-D) for the 94 elements H-Pu. J. Chem. Phys..

[36] Grimme S., Ehrlich S., Goerigk L. (2011). Effect of the damping function in
dispersion corrected density functional theory. J. Comput. Chem..

[37] Helmich-Paris B., de Souza B., Neese F., Izsák R. (2021). An improved
chain of spheres for exchange algorithm. J.
Chem. Phys..

[38] Neese F. (2023). The SHARK
Integral Generation and Digestion System. J.
Comput. Chem..

[39] Becke A. D. (1993). Density-functional
thermochemistry. III. The role of exact exchange. J. Chem. Phys..

[40] Lee C., Yang W., Parr R. G. (1988). Development of the Colle–Salvetti
correlation-energy formula into a functional of the electron density. Phys. Rev. B.

[41] Weigend F., Ahlrichs R. (2005). Balanced basis sets of split valence, triple zeta valence
and quadruple zeta valence quality for H to Rn: Design and assessment
of accuracy. Phys. Chem. Chem. Phys..

[42] Weigend F. (2006). Accurate Coulomb-fitting
basis sets for H to Rn. Phys. Chem. Chem. Phys..

[43] Noodleman L. (1981). Valence bond
description of antiferromagnetic coupling in transition metal dimers. J. Chem. Phys..

[44] Ruiz E., Cano J., Alvarez S., Alemany P. (1999). Broken symmetry approach
to calculation of exchange coupling constants for homobinuclear and
heterobinuclear transition metal complexes. J. Comput. Chem..

[45] Yamaguchi K., Jensen F., Dorigo A., Houk K. N. (1988). A spin correction
procedure for unrestricted Hartree–Fock and Møller–Plesset
wave functions for singlet diradicals and polyradicals. Chem. Phys. Lett..

[46] Lu N., Lokesh V. B., Chan K. L., Gurumallappa G., Gowda Y., Zeng P.-X., Zheng D., Nawa K., Sato T. J. (2025). Synthetic, structural and magnetic studies of a fluorinated
chloro-bridged Cu­(II) dimeric complex, [((C_2_F_5_CH_2_OCH_2_)_2_py)­CuCl_2_]_2_, with unusual non–covalent interactions. J. Fluorine Chem..

[47] Engberg Å., Staffansson L. (1970). An X-Ray Refinement of the Crystal Structure of Copper­(II)
Chloride Dihydrate. Acta Chem. Scand..

[48] Elakkat V., Chang C. C., Chen J. Y. (2019). The first two examples
of halogen bonding with a sigma hole-donating fluorine in the Csp^3^–F···Osp^3^ interaction from
polyfluorinated trans-dihalo-palladium­(ii) di-substituted pyridine
complexes. Chem. Commun..

[49] Alabugin I. V., Manoharan M., Peabody S., Weinhold F. (2003). Electronic Basis of
Improper Hydrogen Bonding: A Subtle Balance of Hyperconjugation and
Rehybridization. J. Am. Chem. Soc..

[50] Elakkat V., Tessema E., Lin C.-H., Wang X., Chang H.-C., Zheng Y.-N., Huang Y.-C., Gurumallappa, Zhang Z.-Y., Chan K. L., Rahayu H. A., Francisco J. S., Lu N. (2023). Unusual Changes of
C–H Bond Lengths in Chiral Zinc Complexes Induced by Noncovalent
Interactions. Angew. Chem., Int. Ed..

[51] Hirota K., Cox D. E., Lorenzo J. E. (1994). Dimerization of CuGeO3
in the Spin-Peierls State. Phys. Rev. Lett..

[52] Bykov M., Zhang J., Schönleber A. (2013). Spin-Peierls distortions
in TiPO4. Phys. Rev. B.

[53] Shaz M., van Smaalen S., Palatinus L. (2005). Spin-Peierls transition
in TiOCl. Phys. Rev. B.

[54] Pascal P. (1910). Recherches
magnétochimiques. Ann. Chim. Phys..

[55] Pascal P. (1912). Recherches
magnétochemiques. Ann. Chim. Phys..

[56] Pascal P. (1913). Magnetochemical
Investigations III. Ann. Chim. Phys..

[57] Bain G. A., Berry J. F. (2008). Diamagnetic Corrections and Pascal’s Constants. J. Chem. Educ..

[58] Bleaney B., Bowers K. D. (1952). Anomalous paramagnetism of copper acetate. Proc. R. Soc. London, Ser. A.

[59] Johnston D. C., Kremer R. K., Troyer M., Wang X., Klümper A., Bud’ko S. L., Panchula A. F., Canfield P. C. (2000). Thermodynamics of
spin S = 1/2 antiferromagnetic uniform and alternating-exchange Heisenberg
chains. Phys. Rev. B.

[60] Desplanches C., Ruiz E., Rodríguez-Fortea A., Alvarez S. (2002). Exchange Coupling
of Transition-Metal Ions through Hydrogen Bonding: A Theoretical Investigation. J. Am. Chem. Soc..

[61] Guedel H. U., Stebler A., Furrer A. (1979). Direct observation of singlet-triplet
separation in dimeric copper­(II) acetate by neutron inelastic scattering
spectroscopy. Inorg. Chem..

[62] Gudel H. U., Stebler A., Furrer A. (1980). Inelastic neutron scattering from
single crystals of dimeric copper acetate. J.
Phys. C: Solid State Phys..

[72] Momma K., Izumi F. (2011). VESTA 3 for three-dimensional visualization of crystal, volumetric
and morphology data. J. Appl. Crystallogr..

