# Supplementary material for: Formation of Antiferromagnetic Spin Dimer Induced by Orientation Ordering of Water Molecule in Fluorinated Pincered Cu(II) Complexes
Source: Inorg Chem. 2026 Sep 11;65(44):25678–88. doi: 10.1021/acs.inorgchem.6c01062 (PMC13753718; doi:10.1021/acs.inorgchem.6c01062)
Supplement: Supplementary file 1 [file ic6c01062_si_001.pdf]

Supplementary Information for "Formation of antiferromagnetic spin dimer induced by orientation ordering of water molecule in fluorinated pincered Cu(II) complexes"

Kazuhiro Nawa[A,*], *Dongge Zheng*[A], *Katsuki Kinjo*[A], *Chun-Wai Tsang*[A,†], *Pin-Xiang Zeng*[B], *Gurumallappa Gurumallappa*[B], *Norman Lu*[B,**], *Taku J Sato*[C,A]

Institute of Multidisciplinary Research for Advanced Materials, Tohoku University, Sendai, 980-8511, Japan[A], Institute of Organic and Polymeric Materials, National Taipei University of Technology, Taipei, 10608, Taiwan (ROC)[B], Institute for Solid State Physics, University of Tokyo, Kashiwa, 277-8581, Japan[C]

E-mail: knawa@tohoku.ac.jp[*], normanlu@mail.ntut.edu.tw[**]

Powder X-ray diffraction patterns of 3F-CuCl$_2$·H$_2$O and 2FH-CuCl$_2$·H$_2$O at room temperatures

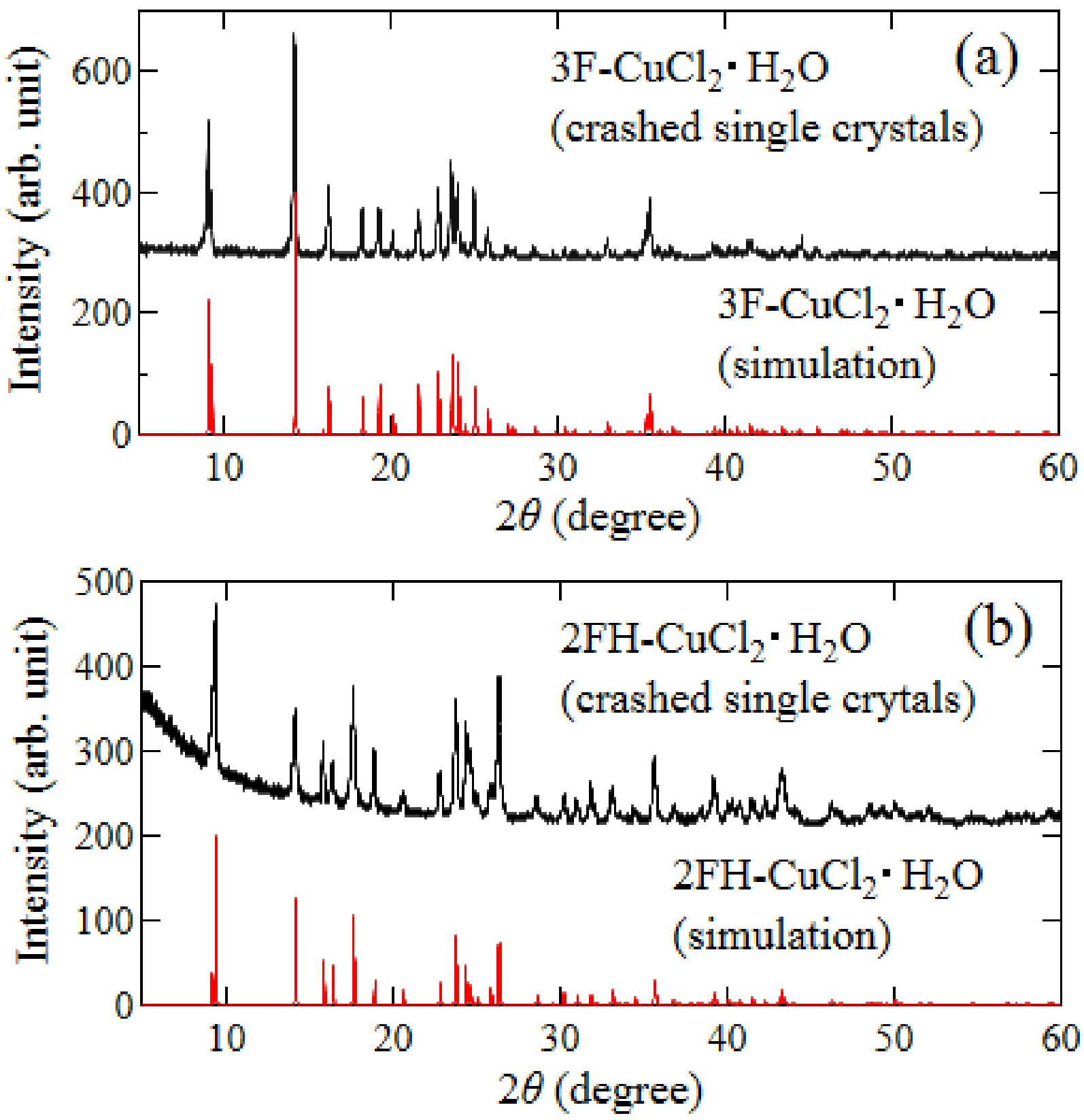

Figure S1 Powder diffraction patterns of (a) 3F-CuCl$_2$·H$_2$O and (b) 2FH-CuCl$_2$·H$_2$O compared with those simulated from the room temperature crystal structure determined in this study.

Crystal structure of $2FH\text{-}CuCl_2{\cdot}H_2O$ at room and at low temperatures

The single crystal X-ray diffraction experiments on $2FH\text{-}CuCl_2{\cdot}H_2O$ reveals the similar molecular and crystal structures to those of $3F\text{-}CuCl_2{\cdot}H_2O$. The crystallographic data and refinement parameters are listed in Table 1 in the main text. $2FH\text{-}CuCl_2{\cdot}H_2O$ crystallizes in the monoclinic space group *C2/c* at 298 K, while it crystallizes in $P2_1/c$ at 100 K. The structure of single molecule is shown in Fig. S2. Its structure is close to that of $3F\text{-}CuCl_2{\cdot}H_2O$, except that a single Cu(II) ion is coupled to a difluorinated pincered $[(CHF_2CH_2OCH_2)_2\text{-py}]$ (2FH-Pin) ligand instead of a $[(CF_3CH_2OCH_2)_2\text{-py}]$ (3F-Pin) ligand. The distribution of $d_{x^2-y^2}$ orbital should be also the same as that of those in $3F\text{-}CuCl_2{\cdot}H_2O$.

At 298 K, intermolecular hydrogen bonding forms a uniform chain of Cu(II) ions with the bond distance of 5.332 Å, as shown in Fig. S3(a). However, there are some differences in the intermolecular hydrogen bonding. Figures S4(a-c) indicate intermolecular H···Cl bonds. The geometry of classical H···Cl hydrogen bond is very similar to those in $3F\text{-}CuCl_2{\cdot}H_2O$, as shown in Fig. S4(a). On the other hand, the improper H···Cl hydrogen bonds are not only present between nearest neighbor molecules but also present between next-nearest neighbor molecules, forming the bond distances of 2.808 Å (Fig. S4(b)). In $2FH\text{-}CuCl_2{\cdot}H_2O$, terminal hydrogen atoms left in ligands can make the improper hydrogen bonding between the next-nearest neighbor molecules active.

At 100 K, the loss of the twofold rotational symmetry allows the dimerization of Cu pairs, as shown in Fig. S3(b). The Cu···Cu bond distance becomes 5.038 and 5.517 Å. In addition, a single type of hydrogen bonds splits into two inequivalent types. The bond length of the improper H···Cl hydrogen bond between nearest neighbor molecules becomes 2.760 and 2.798 Å, and that of the improper H···Cl hydrogen bond between next-nearest neighbor molecules becomes 2.669 and 2.755 Å. In addition, the structural transition is accompanied by the formation of the improper H···F hydrogen bond with the bond length of 2.587, as indicated by dashed red lines in Fig. S4(c). The improper H···F hydrogen bond gets much shorter than 2.805 Å at 298 K, suggesting that it is also effective for driving the orientational ordering of water molecules in $2FH\text{-}CuCl_2{\cdot}H_2O$. It should be noted that the space group of $P2_1/c$ (100 K) corresponds to the maximal subgroup of *C2/c* (298 K) induced by the loss of the twofold rotation symmetry.

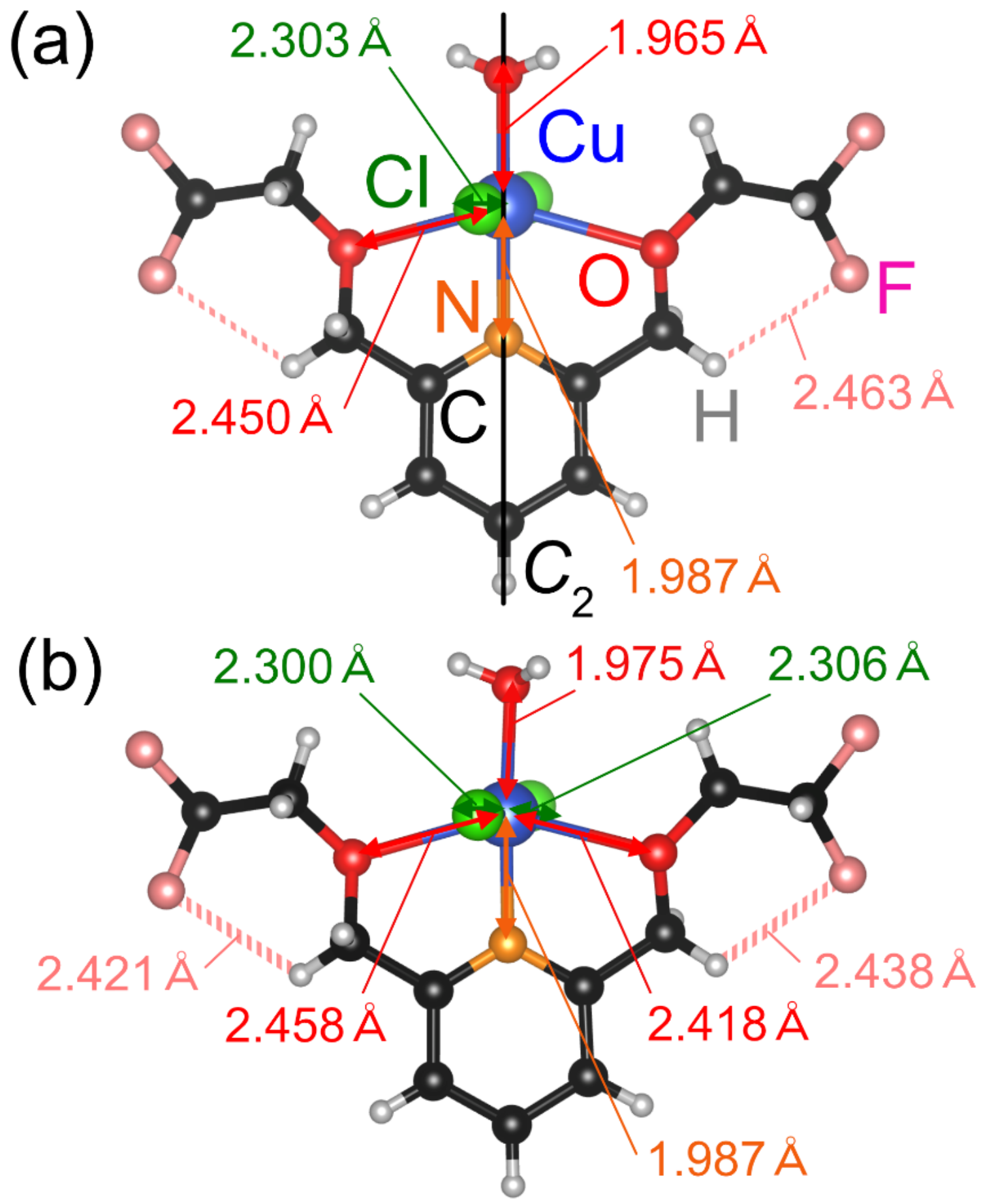

Figure S2 Single monomer molecule of 2FH-$CuCl_2 \cdot H_2O$ at (a) 298 K and (b) 100 K viewed perpendicular to the pyridine ring. The white, pink, black, orange, red, green, and blue circles represent, H, F, C, N, O, Cl, and Cu atoms, respectively.

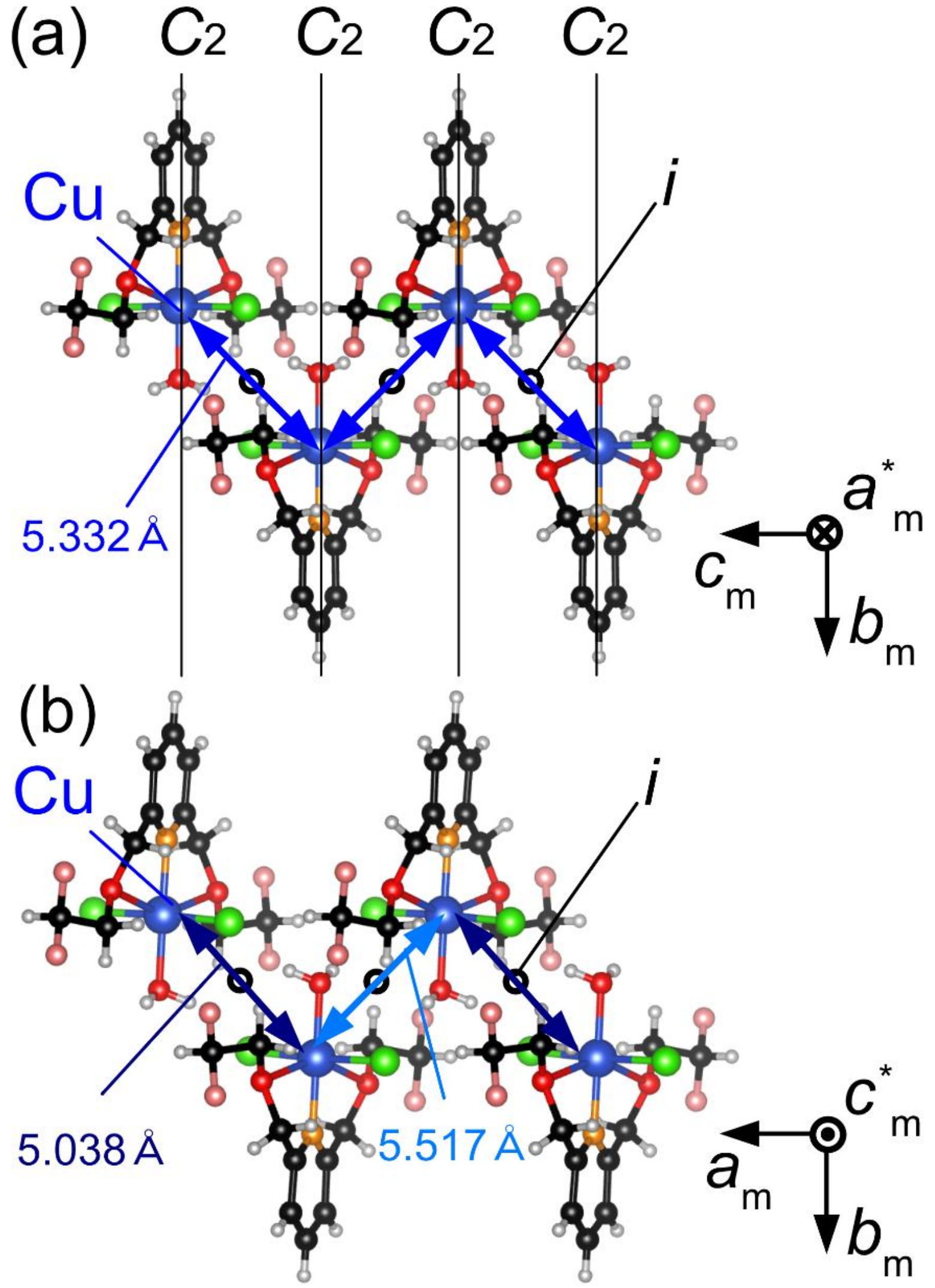

Figure S3 Symmetry operations in of 2FH-$CuCl_2 \cdot H_2O$ for (a) the $C2/c$ structure at 298 K and (b) the $P2_1/c$ structure at 100 K. The structure is viewed along (a) the a^*-axis of the room temperature and (b) c^*-axis of the low-temperature monoclinic structures to show the coupling of Cu (II) ions. The white, pink, black, orange, red, green, and blue circles represent, H, F, C, N, O, Cl, and Cu atoms, respectively.

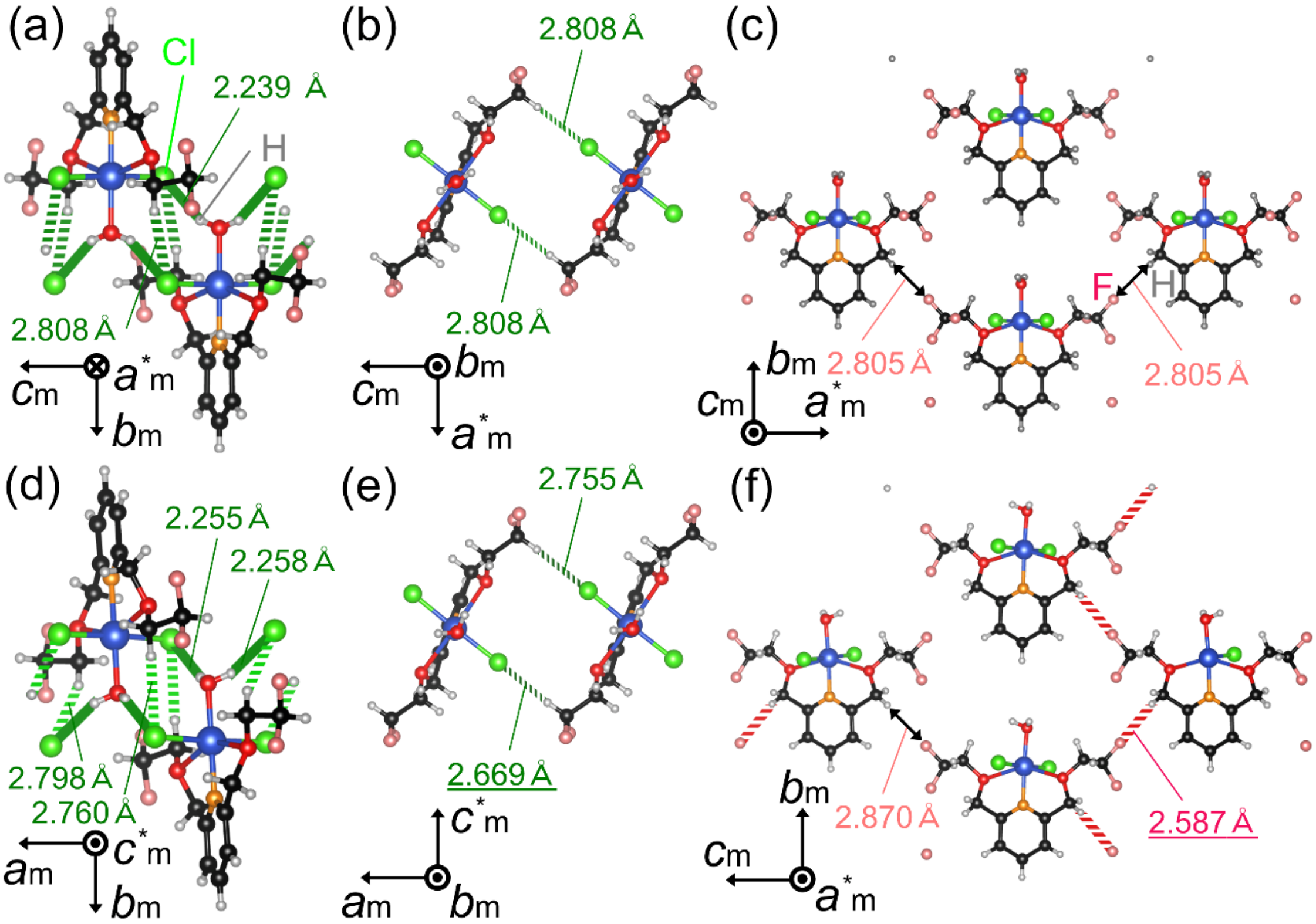

Figure S4 Intermolecular bonds of 2FH-$CuCl_2 \cdot H_2O$ at (a-c) 298 K and (d-f) 100 K. The structure is viewed along the (a) a^*-, (b) b- and (c) c-axis of the $C2/c$ structure and the (d) c^*-, (e) b- and (f) a^*-axis of the $P2_1/c$ structure. The white, pink, black, orange, red, green, and blue circles represent, H, F, C, N, O, Cl, and Cu atoms, respectively.

Estimation of magnetic interactions from magnetic susceptibility

The magnetic susceptibility was fit to the isolated antiferromagnetic spin dimer [1] and alternating chain models [2] to estimate magnetic interactions. The temperature dependence of the magnetic susceptibility is compared with the fitted function in Fig. S5. Both models well explain the whole temperature dependence, suggesting that the interdimer interactions are weak. Small differences between the models are only found at low temperature below 3 K. For instance, in 3F-$CuCl_2 \cdot H_2O$, the magnetic susceptibility expected from the isolated antiferromagnetic spin dimer and alternating chain models is 30~35 and 6~13 % smaller than the experimental value at 2 K, respectively. This is because additional antiferromagnetic interdimer interactions enhance the low-temperature magnetic susceptibility. This effect also appears in the magnetization curve at 2 K, shown in Fig. 6. The lack of interdimer interactions in the isolated dimer model makes the slope of the magnetization curve slightly smaller than that of measured curve in the zero-field limit. The magnetic interactions estimated from the fit is summarized in Table 4.

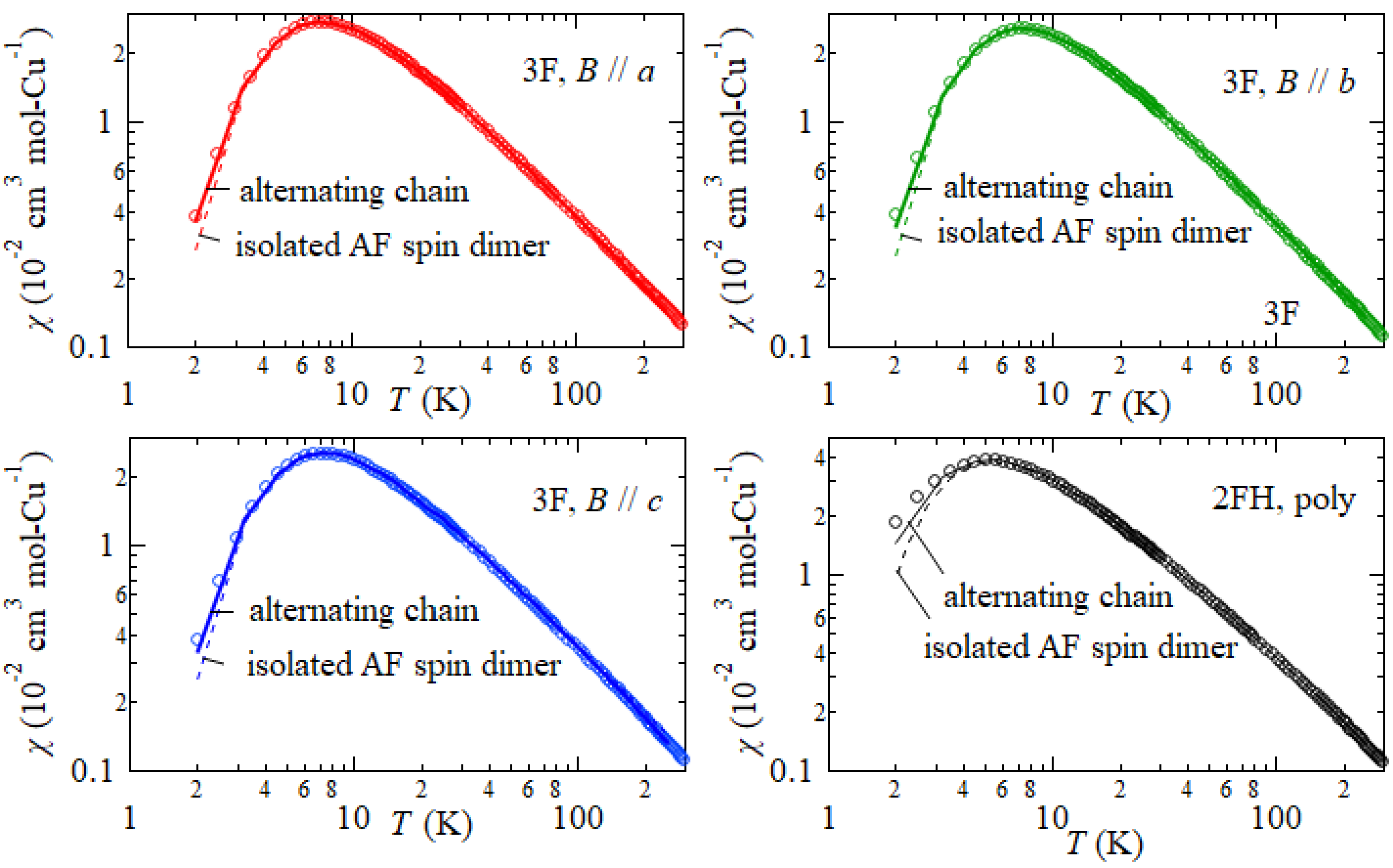

Figure S5 The temperature dependence of the magnetic susceptibility compared with the fit to the isolated antiferromagnetic spin dimer (dashed curves) and alternating chain models (solid curves).

Possible magnetic exchange path through pyridine rings

The DFT calculations suggest the possible exchange path between stacked pyridine rings. To discuss its origin, the atomic distance between 3F-$CuCl_2 \cdot H_2O$ molecules is shown in Figure S6. In molecular magnetic compounds, π-π interactions can be effective if short contact atoms are close enough. The distance between the short contact atoms of the stacked pyridine rings is 3.563 Å (C4-C6) and 3.850 Å (C2-C4). Other systems that include magnetic interactions mediated by π-π interactions have the bond distance typically below 3.4 Å [3-5]. Thus, π-π interactions should not cause sizable magnetic interactions in 3F-$CuCl_2 \cdot H_2O$.

Alternatively, we found that the frontier orbital is mainly distributed along chlorine atoms, as shown in Fig. S7. Magnetic interactions via C-H…Cl path might be possible despite the large Cu-Cu distance of 8.238 Å. The H…Cl bond distances of 2.975, 2.979 and 2.989 Å are still much larger than 2.648 and 2.800 Å formed between the different pair of molecules shown in Fig. 2. In addition, the spin density is distributed to the O atom of the water molecule and the two Cl ligands, and that on the pyridine is much smaller. Thus, we believe that magnetic interactions via C-H…Cl path should not be so strong as the hydrogen mediated magnetic interactions discussed in the main text. It might be only detected as small antiferromagnetic interactions connecting antiferromagnetic dimers. For instance, the fit to the alternating chain model suggests the presence of interdimer antiferromagnetic interactions of -2.4 K, which could be due to magnetic interactions via C-H…Cl path. Almost the same discussion can be applied to 2FH-$CuCl_2 \cdot H_2O$. Further DFT calculations after determining accurate positions of hydrogen atoms are necessary to investigate the exchange path of interdimer magnetic interactions.

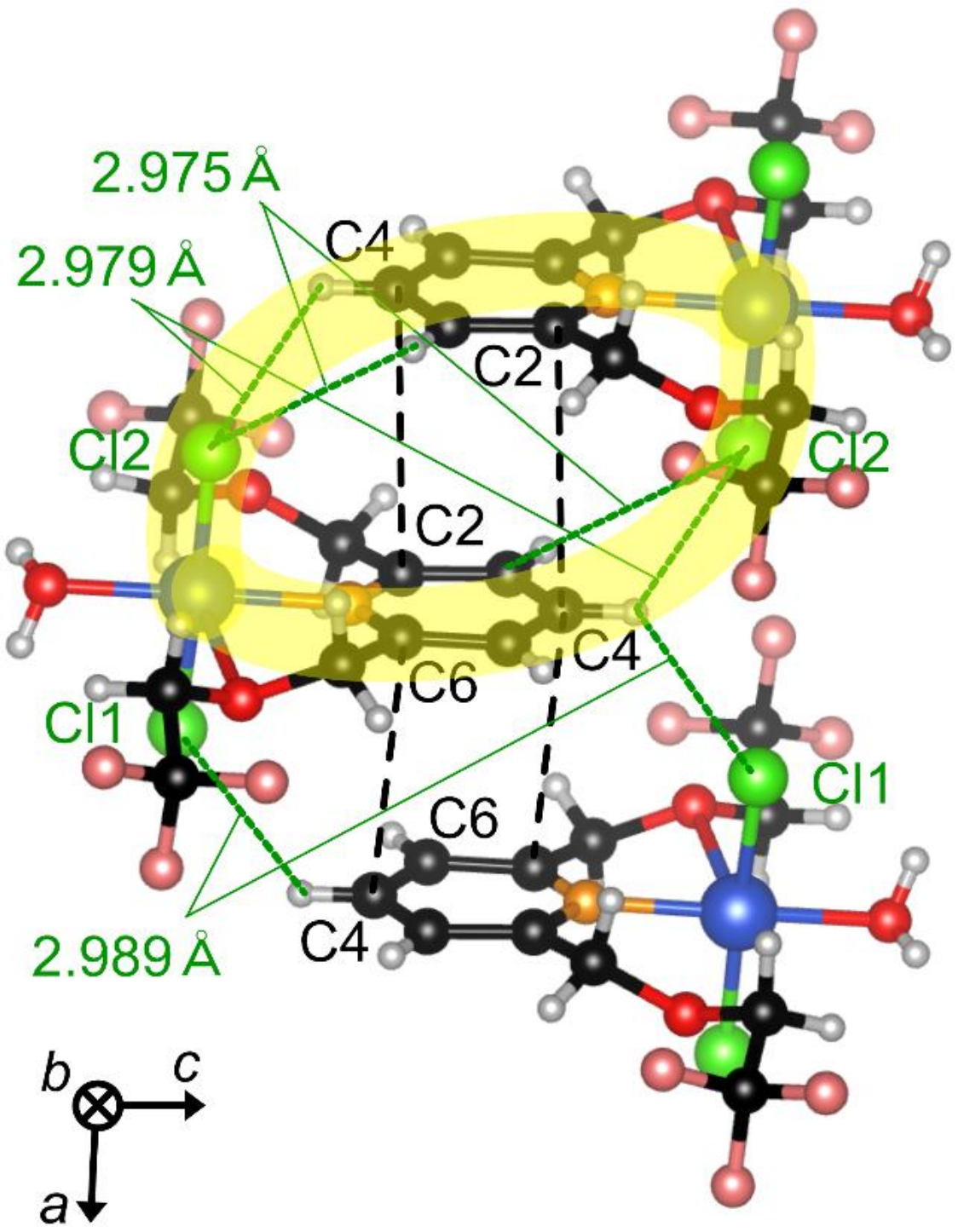

Figure S6 Intermolecular bonds of 3F-$CuCl_2 \cdot H_2O$ at 100 K. The structure is viewed along the *b*-axis of the $P2_1/c$ structure. The white, pink, black, orange, red, green, and blue circles represent, H, F, C, N, O, Cl, and Cu atoms, respectively. Yellow curves are a guide to show the possible exchange path discussed above.

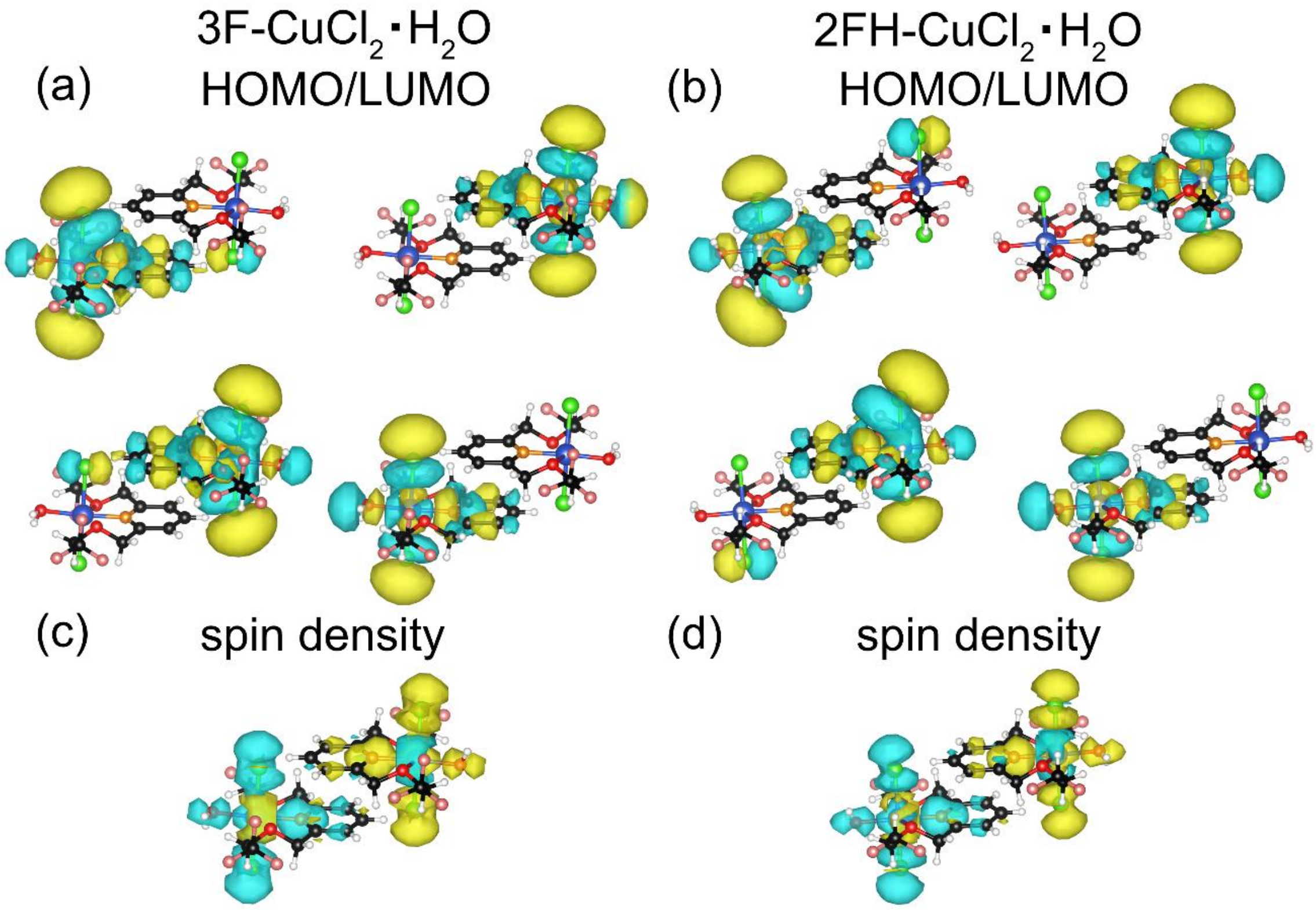

Figure S7 (a,b) Illustration of two highest occupied (HOMO) and two lowest unoccupied orbitals (LUMO) of a pair of (a) 3F-$CuCl_2 \cdot H_2O$ and (b) 2FH-$CuCl_2 \cdot H_2O$ molecule that are coupled with antiferromagnetic interactions via a C–H···Cl path ("stacked pyridine" long in Table 5). (c,d) Illustration of spin density in the pair of (c) 3F-$CuCl_2 \cdot H_2O$ and (d) 2FH-$CuCl_2 \cdot H_2O$ molecules which is the same as those in Fig (a,b). The isosurface values of 0.01 a.u. (a,b) and 0.0005 a.u. (c,d) are used, respectively.

(1) Bleaney., B.; Bowers, K. D Anomalous paramagnetism of copper acetate *Proc. R. Soc. A* **1952**, 214 (1119), 451–465.

(2) Johnston, D. C; Kremer, R. K; Troyer, M.; Wang, X.; Klu¨mper, A.; Bud'ko, S. L; Panchula, A. F; Canfield, P. C Thermodynamics of spin S=1/2 antiferromagnetic uniform and alternating-exchange Heisenberg chains *Phys. Rev. B* **2000**, 61 (14), 9558-9606.

(3) Chi, Y. -; Shi, J. -; Li, H. -; Wei, W.; Cottrill, E.; Pan, N.; Chen, H.; Liang, Y.; Yu, L.; Zhang, Y. -; Hou, C. π–π Stacking, spin density and magnetic coupling *Dalton Transactions* **2013**, 42, 15559-15569.

(4) Venegas-Yazigi, D.; Brown, K. A; Vega, A.; Calvo, R.; Aliaga, C.; Santana, R. C; Cardoso-Gil, R.; Kniep, R.; Schnelle, W.; Spodine, E. *Inorg. Chem.* **2011**, 50 (22), 11461–11471.

(5) Ramirez de Arellano, C.; Escriva, E.; Gomez-Garcia, C. J; Minguez Espallargas, G.; Ballesteros, R.; Abarca, B. *CrystEngComm* **2013**, 15 (10), 1836–1839.
